# Thermostability and in vivo performance of AAV9 in a film matrix

**DOI:** 10.1038/s43856-022-00212-6

**Published:** 2022-11-21

**Authors:** Trang Nguyen Kieu Doan, Matthew D. Le, Irnela Bajrovic, Lorne Celentano, Charles Krause, Haley Grooms Balyan, Abbie Svancarek, Angela Mote, Anna Tretiakova, R. Jude Samulski, Maria A. Croyle

**Affiliations:** 1 The University of Texas at Austin College of Pharmacy, Division of Molecular Pharmaceutics and Drug Delivery, Austin, TX 78712 USA; 2 AskBio 20T.W. Alexander Drive, Suite 110, Durham, NC 27709 USA; 3 Jurata Thin Film, 2450 Holcombe Blvd., Suite J, Houston, TX 77021 USA; 4 Department of Pharmacology, University of North Carolina, 7119 Thurston Bowles Bldg. 104 Manning Dr., Chapel Hill, NC 27599 USA; 5 John R. LaMontagne Center for Infectious Disease, The University of Texas at Austin, Austin, TX USA

**Keywords:** Drug development, Viral vectors

## Abstract

**Background:**

Adeno-associated virus (AAV) vectors are stored and shipped frozen which poses logistic and economic barriers for global access to these therapeutics. To address this issue, we developed a method to stabilize AAV serotype 9 (AAV9) in a film matrix that can be stored at ambient temperature and administered by systemic injection.

**Methods:**

AAV9 expressing the luciferase transgene was mixed with formulations, poured into molds and films dried under aseptic conditions. Films were packaged in individual particle-free bags with foil overlays and stored at various temperatures under controlled humidity. Recovery of AAV9 from films was determined by serial dilution of rehydrated film in media and infection of HeLa RC32 cells. Luciferase expression was compared to that of films rehydrated immediately after drying. Biodistribution of vector was determined by in vivo imaging and quantitative real-time PCR. Residual moisture in films was determined by Karl Fischer titration.

**Results:**

AAV9 embedded within a film matrix and stored at 4 °C for 5 months retained 100% of initial titer. High and low viscosity formulations maintained 90 and 85% of initial titer after 6 months at 25 °C respectively. AAV was not detected after 4 months in a Standard Control Formulation under the same conditions. Biodistribution and transgene expression of AAV stored in film at 25 or 4 °C were as robust as vector stored at −80 °C in a Standard Control Formulation.

**Conclusions:**

These results suggest that storage of AAV in a film matrix facilitates easy transport of vector to remote sites without compromising in vivo performance.

## Plain language summary

Adeno-associated viruses (AAVs) are small viruses that are used to deliver medicines and vaccines. Prior to administration, they are stored in freezers set to very low temperatures and must be discarded if they thaw during transportation to clinics. AAV was embedded in a film to protect the virus during transportation and storage. The virus remained stable for 6 months at room temperature and during shipment from Texas to North Carolina. The ability to store and transport AAV without the need for complex packaging and temperature control will increase global access to vaccines and other medicines that use AAVs for delivery.

## Abstract (web summary)

Doan et al. characterize and assess the stability of film matrix embedded Adeno-Associated Virus 9 (AAV9) vectors during storage and transport at ambient temperatures. High and low viscosity formulations of AAV9 stored at 25 °C maintain titer for 6 months.

## Introduction

For over 4 decades, recombinant adeno-associated viruses (AAVs) have been developed as vectors for gene therapy^1^. There are currently 263 active clinical trials, representing nearly one-third of all trials using a viral vector, in which AAVs are evaluated as therapies for monogenetic, ocular, cardiovascular, lysosomal storage, neuromuscular and infectious diseases^2^. Breakthroughs in recombinant DNA and bioprocessing technologies have allowed AAV to be one of the first viral vectors to gain regulatory approval by the United States Food and Drug Administration (FDA)^3–5^. Despite the fact that AAV was shown to be thermostable at temperatures as high as 56 °C early in its development^6^, each of the current (or formerly) marketed AAV products are formulated as liquid products that are stored and shipped at ultralow temperatures (Table 1)^7–10^. As demonstrated by vaccine distribution during the COVID-19 pandemic, this approach poses a serious barrier to global access of these life-saving therapies.

**Table 1:** Stability profiles of current (or formerly) marketed AAV poducts^7–10^.

	Glybera (alipogene tiparvovec)^7^	Luxterna (voretigene neparvovec-rzyl)^8^	Zolgensma (onasemnogene abeparvovec-xioi)^9, 10^
**Serotype**	AAV1	AAV2	AAV9
**Indication**	Lipoprotein Lipase Deficiency (LPLD)	Biallelic RPE65 mutation-associated retinal dystrophy	Spinal Muscular Atrophy (SMA) with mutations in the SMN1 gene
**Storage**	−25 to −15 °C	−80 °C	−80 °C
**Formulation**	Phosphate buffered saline Sucrose WFI	180 mM NaCl 10 mM sodium phosphate (pH 7.3) 0.001% Poloxamer 188	20 mM Tris (pH 8.0) 1 mM MgCl_2_ 200 mM NaCl 0.005% Poloxamer 188
**Shelf Life**	18 mo −25 to −15 °C	2 years −80 °C	12 mo −80 °C
**Shelf Life** **Thawed** **Other Instructions**	8 h 2–8 °C 8 h RT Protect from light	Prepare and use within 4 h Do not refreeze	Store at 2–8 °C upon receipt. 14 d 2–8 °C Use within 8 h after drawing into syringe Do not shake.

A careful review of the literature reveals a limited number of studies that describe efforts to improve the thermostability of recombinant AAV vectors early in their development as gene therapy vectors with a notable increase as AAV-based products gained regulatory approval^11–18^. While the majority of work has focused on liquid formulation development, lyophilization, a method utilized in the pharmaceutical industry to improve the thermostability of a variety of biological drugs^19,20^, has been shown to promote thermostability of recombinant AAV vectors at ambient temperatures^11,21^. This and other types of drying processes may improve the shelf life of AAV^22^, however, they are not scalable and/or practical for the quantity of vectors needed for current target patient populations^23^. In an effort to improve logistic and economic issues associated with the global distribution of vaccines and other biologic drugs to remote, underdeveloped areas, we established a novel process for stabilizing live viruses in a peelable film that can be shipped and stored at ambient temperature^24^. This approach embeds the virus within a polymeric matrix using inexpensive ingredients and a method that does not require complex instrumentation or training. The small, flat product (Supplemental Fig. 1) would allow large quantities of vectors to be shipped and distributed easily and would require limited infrastructure for long-term storage. However, the process was also originally designed for administration by the oral and/or nasal route as previously illustrated with an adenovirus-based Ebola vaccine^25,26^, so further studies were planned to evaluate AAV performance in a similar film-based platform for use as an injectable product.

The aim of this report is to evaluate the long-term thermostability profile of a recombinant AAV serotype 9 vector containing a luciferase transgene under the control of a hybrid CMV enhancer/chicken β-actin (CBA) promoter within the film matrix. We found that the viscosity and pH of the film formulation, the use of surfactant, and the relative humidity of the environment in which the films are stored significantly impacted the recovery of live virus from the film. Despite efforts to reduce the viscosity of the formulation so that it can be administered systemically, virus embedded within films made from viscous formulations were more stable at 25 °C than that embedded within low-viscosity formulations. Pivotal proof-of-concept in vivo studies in which AAV-containing films that were stored at 4 and 25 °C, shipped from Austin, Texas to Durham, North Carolina, without dry ice/temperature regulation and then rehydrated as intravenous solutions revealed that tissue biodistribution and transgene expression patterns of virus embedded in film followed the same trend as in vitro stability profiles. To our knowledge, this is the first report to compare in vivo performance of an AAV vector after ambient shipping to that of a freshly prepared, frozen vector in a standard formulation.

## Materials and methods

### Materials

Dulbecco’s phosphate buffer saline (DPBS), Trizma base [2-amino-2-(hydroxymethyl)−1,3-propanediol] (Tris), and *N*-(2-hydroxyethyl)piperazine-*N* ’-(2-ethanesulfonic acid) (HEPES) were purchased from Sigma-Aldrich (St. Louis, MO). Sorbitol solution (70% USP) was purchased from Spectrum Chemicals (Gardena, CA). Glycerol [USP grade], glycine, ammonium persulfate, tetramethylethylenediamine, and Aqualine Complete 5 solvent were purchased from Thermo Fisher Scientific Chemicals (Fairlawn, NJ). Dulbecco’s modified Eagle’s medium (DMEM) and 0.25% Trypsin EDTA were purchased from Mediatech (Manassas, VA). Penicillin (10,000 IU)/streptomycin (10,000 µg/ml) and fetal bovine serum (qualified FBS, Mexico Origin) were purchased from Gibco Life Technologies (Grand Island, NY). Ultrapure Protogel (30% acrylamide and 8% bis-acrylamide) was bought from Natural Diagnostics (Atlanta, GA). Poly (maleic anhydride-alt-1-octadecene) substituted with 3-(dimethylamino)propylamine, poly (maleic anhydride-alt-1-decene) substituted with 3-(dimethylamino) propylamine, poly (maleic anhydride-alt-1-tetradecene) substituted with 3-(dimethylamino) propylamine were obtained from Anatrace (Maumee, OH). Hydranal^TM^ Formamide anhydrous solvent was purchased from Fluka Honeywell Research Chemicals (Morris Plains, NJ). Methanol, 99.8% extra dry, was obtained from Acros Organics (Fair Lawn, NJ). Oligonucleotide primers were custom synthesized by Sigma Life Science (Woodlands, TX). All other chemicals were of analytical reagent grade and purchased from Thermo Fisher Scientific (Pittsburgh, PA) unless specified otherwise. The authors agree that all reasonable requests for materials utilized in the studies summarized in this manuscript will be fulfilled and will require the establishment of a Materials Transfer and/or License Agreement between interested parties and the University of Texas at Austin, Jurata Thin Film, and/or AskBio.

### AAV production and purification

The AAV9 CBA-Luc vector was prepared by triple plasmid-based transient transfection of a suspension of a proprietary HEK293 cell line (Pro10^TM^, AskBio, Durham, NC). Cells were expanded in a series of shake flasks to seed a 50 L stirring production bioreactor. Cells were then transfected with a cocktail consisting of pXX680 (Adenovirus helper plasmid), AAV helper plasmid (rep2/capX), the transgene-containing vector plasmid (TR plasmid), and transfection reagent. Cells were harvested between 48–72 h post-transfection. Cells were chemically lysed to release the viral vector, treated further to remove cellular DNA and RNA, and debris clarified by filtration. Intact rAAV particles were further purified by affinity capture chromatography. Full capsids were selected from empty capsids by iodixanol density gradient centrifugation. The purified bulk virus from the iodixanol centrifugation step was further purified and concentrated using a quaternary amine chromatography resin. Contaminant proteins that do not bind to the column were removed in the flow through and from column wash steps. The column eluate containing purified rAAV vector was concentrated and diafiltered into Standard Control Formulation (phosphate-buffered saline, 350 mM NaCl, 5% Sorbitol, 0.001% Pluronic F68 (pH 7.4)). The formulated, purified rAAV solution was then filtered into sterile containers and stored at −80 °C until use.

### Cell culture

HeLa RC32 cells (CRL-2972, ATCC, Manassas, VA, passage 16–20) were maintained in 100 mm culture dishes (Corning Falcon, Corning, NY) containing Complete Media (DMEM, 10% FBS, 1% of penicillin (10,000 IU)/streptomycin (10,000 µg/ml). Cells were seeded at 1 × 10^4^ cells per well in 96-well plates (Corning Falcon) 72 h prior to use in limiting dilution and cytotoxicity assays.

### Formulation screening

Formulations consisting of 1.5% polymer base, 2% sugar, and 1% surfactant were prepared in bulk with various solvents and homogenized using a rotary mixer prior to the addition of AAV at a concentration of 1 × 10^12^ virus genomes (v.g.)/ml. Films were dispensed into 1 ml unit dose molds using an E3 Repeater pipette (Eppendorf, Hauppauge, NY) and dried under constant airflow, ambient (21.5 ± 1.5 °C, 1 atm, 40–55% relative humidity), and aseptic conditions. Temperature and humidity were monitored during the film-forming process using an Ambient Weather WS-3000-X5 Wireless Thermo-Hygrometer (Chandler, AZ). pH was monitored during the film-forming process by the addition of a Universal pH Indicator (Fisher Chemicals, Hampton, NH) to placebo films, visual inspection, and comparison to a reference Indicator Solution Color Chart provided with the reagent. Once dry, films were peeled and placed in Infection Media (25 mM HEPES in DMEM) containing 3.2 × 10^8^ particles of first-generation adenovirus serotype 5 at 37 °C. The resulting solution was serially diluted in tenfold increments and used to infect HeLa RC32 cells as part of an in vitro transduction assay. The amount of live virus retained in the film after drying was calculated using Eq. (1) below:

1 $${{{{{\rm{Percent}}}}}}\,{{{{{\rm{Recovery}}}}}}=\frac{\log (infectious\,titer\,t=n)}{\log (infectious\,titer\,t=0)}\times 100$$

where *t* = n is the virus genome copy number equivalent to luciferase expression (in RLU) obtained from AAV in a film on a given day (n) after storage/treatment and *t* = 0 is the virus genome copy number equivalent to luciferase expression obtained from AAV in the same formulation the day it was initially prepared. Additional information about formulation compositions can be found in Supplemental Table 1.

### In vitro infectious titer assay

Fifty microliter aliquots of rehydrated, serially diluted virus-containing films were added to triplicate wells of 96-well plates of confluent HeLa RC32 cells. Two hours later, cells in each well were fed with 50 µl of Complete Media. Seventy-two hours later, 50 µl of media was removed and replaced with 50 µl of the luciferase assay substrate of the ONE-Glo™ Luciferase Assay System (Promega, Madison, WI) in half of the plates that were infected with AAV. Luciferase intensity for each well was read using a microplate reader (GloMax-Multi+ Detection System, Promega). Cells in the remaining plates were washed twice with DPBS and trypsinized for assessment of internalized virus genome copies by qPCR. Cells were resuspended in DPBS and viral DNA was extracted from each well using QIAamp® DNA mini kits (Qiagen, Germantown, MD) according to the manufacturer’s instructions. For each experiment, a standard curve consisting of log RLU vs log virus genomes was created with linearity ranging from 5 × 10^6^ to 5 × 10^9^ vg per well.

### Quantitative real-time PCR (qPCR)

The number of virus genomes in cell and tissue samples was assessed using a qPCR assay specific for the luciferase sequence (forward primer 5′-TGCACATATCGAGGTGGACATC-3′, reverse primer 5′-TGCCAACCGAACGGACAT-3′ (Sigma-Aldrich, St. Louis, MO) and Taqman MGB probe 6FAM-CTTACGCTGAGTACTTCG-MGBNFQ (Applied Biosystems, CA) with a ViiA7 Real-Time PCR system (Applied Biosystems, Waltham, MA) under the following cycling conditions: 95 °C for 30 seconds, followed by 40 cycles of 95 °C for 5 s followed by 60 °C for 30 s. PCR reactions, 25 μl final volume, consisted of 5 µl sample or standard, 0.3 μM of each primer, 0.1 μM of the labeled probe, and 12.5 μl of 2× TaqMan Fast Advanced Universal PCR master mix (Thermo Fisher Scientific Baltics (Vilnius, Lithuania)) in MicroAmp Fast 96-well optical reaction plates (Applied Biosystems). Standard curve samples were prepared from AAV9 plasmids in the range of 1 × 10^3^ to 1 × 10^9^ vg/well. All samples were run in triplicate.

### Characterization of films: dissolution

Films were placed in sterile chambers equipped with a stir bar rotating at 60 rpm and one milliliter of DPBS prewarmed to 37 °C. Samples (10 μL) were collected on a schedule of 0, 5, 10, 15, 20, 25, 30, 45, 60, 75, 90, 105, and 120 min and replaced by an equal amount of DPBS to maintain a constant volume. The transduction efficiency of AAV present in each sample was determined by luciferase transgene expression and virus genomes present in dissolution media. Samples were also taken of the virus in liquid film formulations that did not undergo drying and of the virus in DPBS alone, which were also stirred at 37 °C. Results from these controls were used to normalize data for changes in titer due to temperature, physical agitation, and formulation effects. The rate of release for a given preparation was determined by taking the slope of the linear phase (from *t* = 0 to *t* = 30 min) of the virus genomes vs the time graph for each replicate of a given formulation. This value is reported in virus genomes/minute. The average rate of release was calculated as the average of individual slopes obtained from release profiles generated for five individual films of each formulation tested.

### Characterization of films: freeze/thaw resistance

Films containing AAV at a concentration of 1 × 10^12^ v.g./ml were prepared in bulk, dried under ambient, aseptic conditions, peeled, and packaged individually in Ziploc-like particle-free bags (American Cleanstat, Irvine, CA) that were placed in groups of 5 in heat sealed Ziploc-like foil bags (Ted Pella, Redding, CA). Bags were placed at −80 °C for at least 24 h and then thawed at 20 °C on the laboratory bench for 90 min to complete a single freeze-thaw cycle. Films collected after 0, 1, 4, 8, 12, and 16 freeze-thaw cycles were rehydrated with Infection Media prewarmed to 37 °C, serially diluted, and used to infect HeLa RC32 cells as part of an in vitro transduction assay. AAV prepared in the Standard Control Formulation was included to compare the amount of virus retained within the film matrix with each freeze-thaw cycle. It was also a very important physical control as it served as an indicator to determine when each cycle (freezing and thawing) was complete. Films showed no change in the physical state during the film-forming process as they did not become brittle nor demonstrate any “sweating” or liquidification properties throughout the entire freeze-thaw process.

### Characterization of films: moisture content

Residual moisture content was determined in films by Karl Fisher volumetric titration^27^. Films were weighed (mext) and placed in EP™ VOA Glass Vials with sealed septa (Thermo Fisher Scientific, Waltham, MA). One milliliter of Extraction Solvent (1:1 volume ratio of anhydrous formamide and extra dry methanol) was weighed and added to the vial through the septa (msol). Vials were placed in a 37 °C water bath and gently agitated until the films were fully dissolved. The solution of the rehydrated film was withdrawn from the vial and dispensed in the titration vessel of a V10S Volumetric Titrator (Mettler Toledo, Columbus, OH) and the moisture content was recorded (C). Residual moisture content for each film was then calculated using Eq. (2) below, where B represents the moisture content of the solvent used to dissolve the films.

2 $${{{{{\rm{Residual}}}}}}\,{{{{{\rm{Moisture}}}}}}\,( \% )=C \times \left(\frac{msol+mext}{mext}\right)-\left(\frac{B \,\times msol}{mext}\right)$$

### In vivo assessment of AAV performance

All procedures were approved by the Institutional Animal Care and Use Committee of Mispro Biotech Services (protocol 2021-ASK-03) and are in accordance with the guidelines established by the National Institutes of Health for the humane treatment of animals. Male B6(Cg)-Tyrc-2J/J (B6 Albino) mice (8–10 weeks) were obtained from the Jackson Laboratory (Bar Harbor, ME) and were housed in a temperature-controlled, 12-h light-cycled facility. Mice were given free access to 5053 irradiated rodent feed (LabDiet, St. Louis, MO) and reverse osmosis-filtered water via an automatic system throughout each study. Male mice were utilized for this study as the estrus cycle in female mice negatively affects AAV transduction^28^.

### Vector administration

Packaged films were shipped overnight via courier at ambient temperature without refrigeration/dry ice from The University of Texas at Austin to Durham, NC. During transit, the environment within sealed packages was monitored with Cryopak iMini USB single-use (temperature only) or Elitech Tlog B100H Digital (temperature and relative humidity) data loggers. Upon arrival, films were either stored at ambient temperature (25 °C) or 4 °C prior to use. Virus in frozen Standard Control Formulation was shipped from Texas to North Carolina at the same time as films in a separate package with dry ice. This control preparation was thawed only once prior to administration to animals. Prior to rehydration, films were equilibrated to room temperature on the lab bench for 20–30 min before they were brought to a biological safety cabinet. In the cabinet, the foil bag was opened and individual films in particle-free bags were removed. Films were removed from bags with sterile forceps and placed in sterile 1.5 ml microcentrifuge tubes containing one milliliter of warm (37 °C) sterile saline. Tubes were gently swirled by hand until the film could not be visually detected. Solutions were mixed by slowly pipetting up and down 5–10 times to avoid creating bubbles. Solutions were then diluted to target doses of 1 × 10^10^ and 1 × 10^11^ vg/mouse with sterile saline. Bioluminescent images of animals were captured using the IVIS® Spectrum imaging system (PerkinElmer, Billerica, MA) prior to vector administration and 8, 15, 22, and 29 days after administration of vector by tail vein injection. Mice were humanely euthanized on day 30. Organs were harvested and bioluminescent images of whole organs were captured. Organs were then divided for assessment of transgene expression and biodistribution of vector genomes by qPCR. All tissues were stored at −80 °C prior to analysis.

### In vivo transgene expression

The distribution of the luciferase transgene was determined by the level of bioluminescence from living animals after substrate injection. At each timepoint, animals were given a 150 mg/kg split dose of IVISbrite™ D-Luciferin Potassium Salt (PerkinElmer) in Phosphate-Buffered Saline pH 7.4 (Thermo Fisher Scientific) by intraperitoneal injection. Image acquisition and analysis were performed using the IVIS® Spectrum and the manufacturer’s software (PerkinElmer).

### Statistics and reproducibility

Statistical analysis of data was performed using Prism software (GraphPad Prism v.9.4.1, San Diego, CA). The following formula was used to determine the minimum number of replicates needed per group in order to determine statistically significant differences between different formulations or treatment groups:

3 $${{{{{\rm{n}}}}}}=\frac{2({{{{{\rm{Z}}}}}}{{{{{\rm{\alpha }}}}}}+{{{{{\rm{Z}}}}}}{{{{{\rm{\beta }}}}}})\,{{{{{\rm{\sigma }}}}}}2}{\Delta 2}$$

Where n is the sample size, Z_α_ is the value for the two-tailed α, Z_β_ is the value for the one-tailed β, σ is the sample standard deviation, and Δ is the smallest difference between the two study groups thought to be important. Values used in the equation were: Z_α_ = 1.96, Z_β_ = 1.282, and σ = 0.1. The value for Δ varied between in vitro and in vivo assays. Paired *t*-tests were used to evaluate significant differences between the two treatment groups. For studies containing more than two unique treatments, ANOVA was utilized to determine statistical significance. Dunnett’s multiple comparison tests were used to compare a number of groups with a control group, while Tukey post hoc tests were utilized to compare every two groups in an entire set of data.

### Reporting summary

Further information on research design is available in the Nature Portfolio Reporting Summary linked to this article.

## Results

### Assessment of short-term stability of recombinant AAV in a standard control formulation

**Fig. 1:**
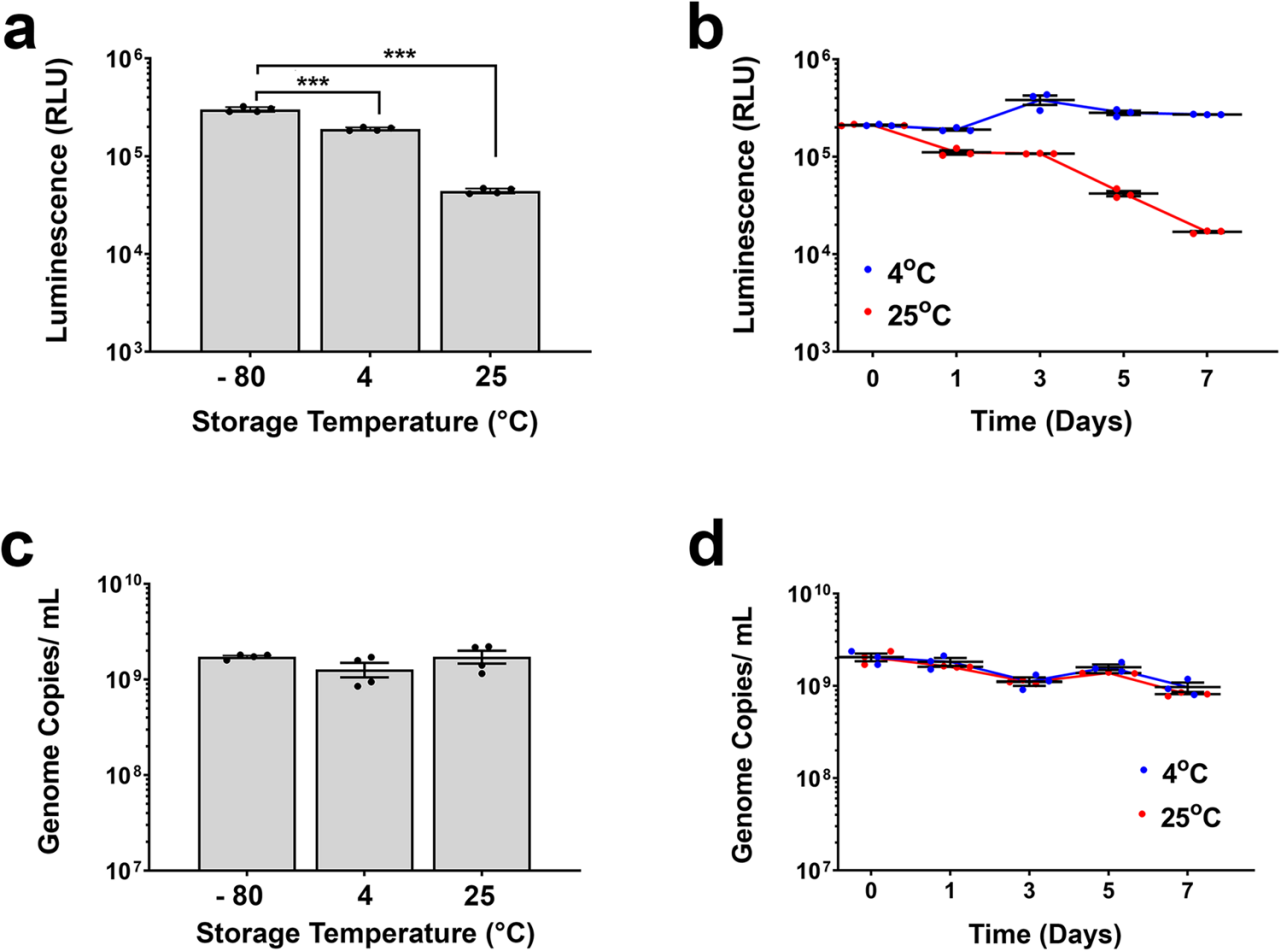
Stability profile of recombinant AAV for 48 h and 7 days at 4 and 25 °C prior to reformulation in film matrix. AAV expressing the luciferase transgene was placed in Standard Control Formulation (phosphate-buffered saline, 350 mM NaCl, 5% Sorbitol, 0.001% Pluronic F68, pH 7.4). Replicate vials were placed at 4 and 25 °C. Three vials per timepoint were collected and assayed for transgene expression by serial dilution on HeLa RC32 cells and luciferase expression was assessed 72 h after infection (Panels **a**, **b**). DNA from replicate plates of cells were harvested and virus genome copies were assessed by quantitative real-time PCR (Panels **c**, **d**). Data from the vector stored in the same formulation at −80 °C is included as a point of reference and serves as the comparator for statistical significance. In all panels, data represents the average ± the standard error of the mean for triple replicates collected at each timepoint. ***p < 0.001, one-way ANOVA with Dunnett’s post hoc test.

Prior to studying the stability of recombinant AAV9 within a film matrix, a small-scale stability study was initiated to evaluate changes in potency when the vector was placed in a Standard Control Formulation (phosphate-buffered saline, 350 mM NaCl, 5% Sorbitol, 0.001% Pluronic F68, pH 7.4) and stored at 4 and 25 °C. A significant drop in infectious titer was detected in cells transduced with vector stored at 25 °C for 48 h with respect to vector stored in the same formulation at −80 °C (3 ± 0.2 × 10^5^ to 4.42 ± 0.2 × 10^4^ RLU, *p* = 0.00003, Fig. 1a). This trend continued for 7 days as luciferase levels fell from 2.1 ± 0.02 × 10^5^ to 1.7 ± 0.02 × 10^4^ RLU while luciferase expression from same preparation stored at 4 °C remained constant at 2.8 ± 0.13 × 10^5^ RLU throughout the same time period (Fig. 1b). In sharp contrast, the number of virus genomes present in cells infected with the same preparations stored at both 4 and 25 °C did not correlate with changes in transgene expression (Fig. 1c, d). Thus, luciferase expression was identified as the more sensitive assay for assessment of the amount of active vector present within the thin-film matrix for optimization and long-term stability studies.

### Impact of film components on recovery of live AAV during the film-forming process

**Fig. 2:**
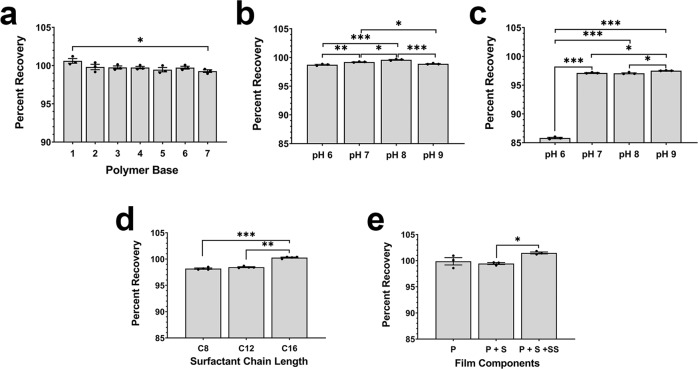
pH and film components impact recovery of a live vector from film matrix. **a** Polymer base compositions with decreasing viscosities (1 highest, 7 lowest) were screened for their film-forming capacity and preservation of AAV transduction efficiency during drying. **b** Recovery of AAV from films prepared with polymer base buffered to different pH after drying was evaluated using an infectious titer assay. **c** The impact of the pH of the polymer base was more evident after films were stored at room temperature for 14 days. **d** Films prepared with surfactant containing a side chain of 8 (C8), 12 (C12), or 16 (C16) carbons revealed that the C16 compound prevented the loss of vector during the drying process. **e** Stepwise addition of polymer base (P), sugar (S), and surfactant (C16, SS) revealed that all three components work in concert to prevent loss of vector during the film-forming process. In panels **b** and **c**, formulations of pH 7–9 were prepared with 10 mM Tris buffer and the pH was adjusted accordingly. The pH 6 formulation was prepared with 100 mM citrate buffer. In each panel (**a**–**e**), data represent the average ± the standard error of the mean of three films per condition and statistical significance between all treatment groups determined by one-way ANOVA with Tukey’s multiple comparison tests with notations of *p < 0.05, **p < 0.01, ***p < 0.001 for significance. Descriptions of formulation compositions utilized in these studies can be found in Supplemental Table 1.

The formulation previously optimized for use with a recombinant adenovirus^24^ was highly viscous (4000 cp)^29^. While this is acceptable for administration by the oral and nasal route, it is physiologically incompatible with intravenous administration, the manner by which many AAV-based and other vectors are currently administered^30–32^. Seven different polymers with viscosities ranging from 15–4000 cp were screened for their ability to maintain AAV potency during the film-forming process. The original potency was retained by polymers with viscosities at or above 100 cp during the film-forming process (Formulations 1–6, Fig. 2a and Supplemental Table 1). Films prepared from the lowest viscosity polymer (Formulation 7) were difficult to peel and, as a result, demonstrated the most significant drop in potency after drying (*p* = 0.0252). The polymers of the highest viscosity (Formulation 1, 4000 cp) and lowest viscosity (Formulation 6, 100 cp) that maintained AAV viability were selected for additional study.

Changes in environmental pH are detrimental to the recovery of recombinant viruses during drying in a lyophilized or thin-film platform^11,24^. Films prepared with polymer in a buffer of pH 8 offered a significant improvement in preserving AAV titer during the film-forming process with respect to those prepared in buffers of pH 6, 7, and 9 (*p* = 0.00006, 0.0128, and 0.0003, respectively, Fig. 2b). Storage of films prepared with each buffer at room temperature (25 °C) for 14 days revealed the impact of pH on long-term stability as films prepared with polymer base in a buffer of pH 6 retained 85.8 ± 0.1% of the original titer while ~97% of the original titer was retained in formulations prepared with base polymer alone in buffers of pH 7–9 (Fig. 2c). Surfactants are often included in film-based formulations to improve dispersion of a medicinal agent throughout the matrix, the release of agent during dissolution and to improve the thermostability profile of live viruses at ambient temperatures^24,33–35^. Prior work demonstrated that an amphipathic surfactant with a 16-carbon side chain preserved adenovirus infectivity during the film-forming process, while those with shorter side chains did not^35^. This was also the case with the AAV9 vector, as the same compound (C16) fully preserved AAV potency during the drying process, while a compound containing an 8-carbon side chain (C8) maintained 98.2 ± 0.1% of the original titer (Fig. 2d). Inclusion of this surfactant (SS) in the film base (P) with a sugar (S) improved recovery of an active vector from 98% (base alone, P) to 100% (P + S + SS, Fig. 2e). Taken together, formulations containing either high or low viscosity polymer base prepared at pH 8 with surfactant and sugar in the matrix were utilized for the remainder of the study.

### Impact of formulation on AAV9 release characteristics from the film matrix

**Fig. 3:**
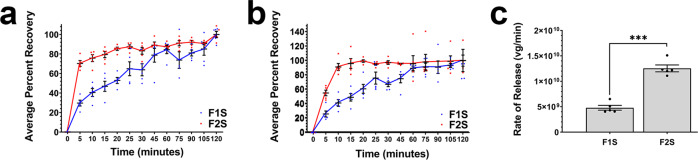
Viscosity of the polymer matrix controls the rate of release of AAV from the film. **a** Cumulative release profile of films prepared with high (F1S, blue line) and low (F2S, red line) viscosity polymers as determined by transgene expression. Films containing 1 × 10^12^ virus genomes were placed in warmed (37 °C) phosphate-buffered saline with gentle agitation and samples were collected over a period of 2 h. Infectious titer of vector released in dissolution medium was determined by infection of HeLa RC32 cells and assessment of luciferase expression. **b** Cumulative release profile of virus genomes from films as determined by quantitative real-time PCR. Curves followed a similar trend as those based upon transgene expression. **c** Average release rate of AAV genomes from film formulations. Data collected during the dissolution of each film was normalized with that generated from the vector placed in the correlating liquid formulations to account for any loss attributable to agitation and extended exposure to heat. In each panel, data represents the average ± the standard error of the mean of five films per condition. In Panel **c**, ***p < 0.001, two-tailed Student’s *t*-test.

The release profile for the AAV9 vector from each formulation was characterized by assessing the transgene expression of the live vector (Fig. 3a) and the number of virus genomes (Fig. 3b) in samples collected over a 2 h period. Within 5 min, 70.3 ± 3% of the total amount of vector embedded within the film was released from the low-viscosity film matrix (F2S), while only 29.7 ± 3.5% of the total dose was released from high-viscosity films (F1S) at the same timepoint (Fig. 3a). Approximately 80% of the total dose was released from the high-viscosity film after 45 min while the same amount of vector was released from the low-viscosity matrix within 15 min. A similar trend was observed for profiles generated on the same samples using quantitative real-time PCR, with 74.7 ± 5.7% of the total number of virus genomes released within 45 min from the high-viscosity matrix, while 95.8 ± 6.8% of the total dose was released from the low-viscosity film within 15 min (Fig. 3b). Linear regression of data collected in the first 30 min of each dissolution curve revealed that the low-viscosity formulation released the AAV vector at a rate of 1.2 ± 0.02 × 10^10^ vg/min which was three times that observed from the high-viscosity preparation (4.4 ± 0.2 × 10^9^ vg/min, Fig. 3c).

### Impact of residual moisture content and environmental humidity on recovery of the vector from film matrix

**Fig. 4:**
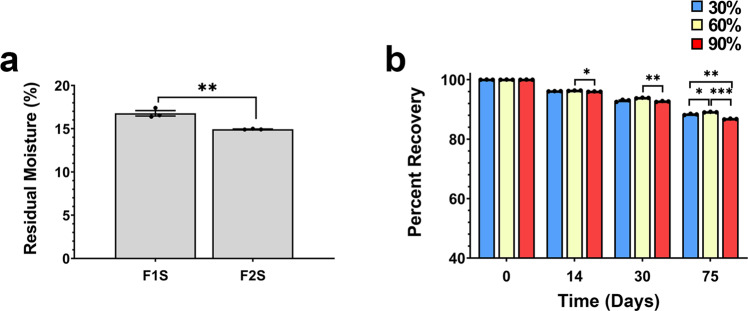
Formulation and environmental humidity influence residual moisture content and long-term stability of AAV within the film matrix. **a** Residual moisture in high (F1S) and low (F2S) viscosity films after drying. Data represent the average ± the standard error of the mean for three replicates for each formulation. **p < 0.01, two-tailed Student’s *t*-test. **b** Sixty percent relative humidity (yellow bars) maintains AAV stability within the film matrix. Films containing 1 × 10^12^ virus genomes were prepared in batches of the high-viscosity formulation (F1S) and stored at 25 °C in different environments with respect to humidity. Films (3 per timepoint and storage condition) were rehydrated with warm culture media and infectious titer was determined by limiting dilution. *p < 0.05, **p < 0.01, ***p < 0.001, two-way ANOVA with Tukey’s post hoc multiple comparison tests.

The amount of water that remains within a dried film dictates both the physical properties of the film and the thermostability profile of viruses in the dried state^11,36^. Low-viscosity films (F2S) retained significantly less water than those prepared with the high-viscosity polymer (F1S, 14.9 ± 0.01 vs 16.8 ± 0.3%, *p* = 0.0043, Fig. 4a). In prior work with adenovirus, we found that thermostability of virus at elevated temperatures was influenced by the relative humidity of the environment in which films were stored^24^. Thus, films prepared with the F1S formulation were packaged and stored in stability chambers set at 25 °C with varying levels of relative humidity (RH). After 14 days, at 90% RH, infectious titer dropped by 4.4 ± 0.01% and by 14.3 ± 0.13% at day 75 (Fig. 4b). Potency in films stored at 30% RH dropped by 12.7 ± 0.22% at end of the study. Films stored at 60% RH lost less than 10 ± 0.21% of the initial dose during the 75-day period, suggesting that all remaining long-term stability studies be performed under these conditions.

### Long-term stability and resistance to external stressors

**Fig. 5:**
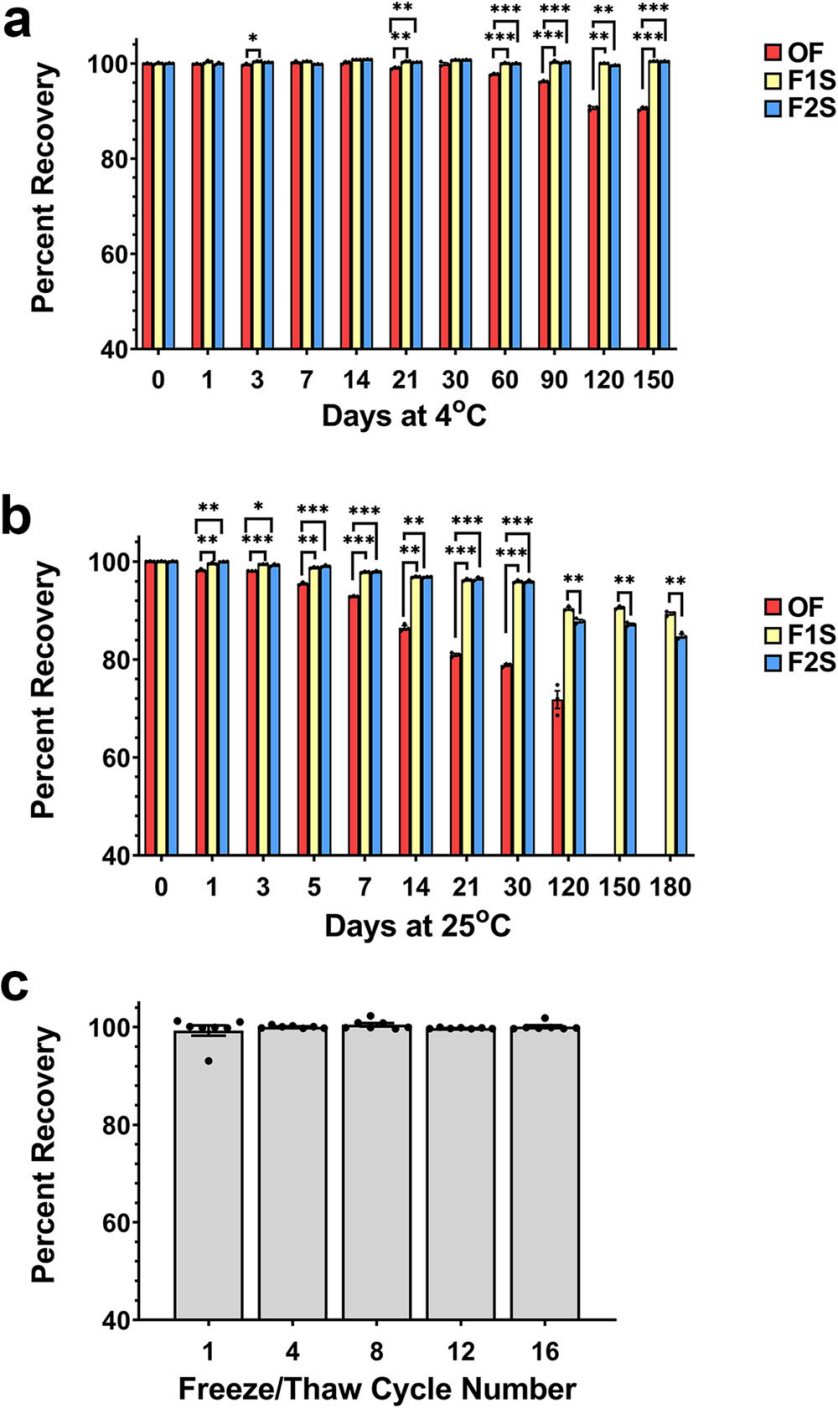
Optimized film matrices improve AAV stability. Films containing 1 × 10^12^ virus genomes were prepared in batch and either stored in controlled environmental chambers held at 4 °C/40–50% RH (Panel **a**) or 25 °C/60% RH (Panel **b**) or subjected to a series of 16 freeze-thaw cycles (Panel **c**). Replicates (at least 3 per timepoint) were reconstituted and live AAV concentration was assessed by a standard infectious titer assay. In Panels **a** and **b**, the Standard Control Formulation (OF, red bars) consisted of phosphate-buffered saline, 350 mM NaCl, 5% Sorbitol, and 0.001% Pluronic F68 (pH 7.4). In each panel, data represents the average ± the standard error of the mean of three replicates per timepoint or condition. In Panels **a** and **b**, significant differences between the F1S (yellow bars) and F2S (blue bars) formulations with respect to the standard control formulation (OF) were evaluated by two-way ANOVA with Dunnett’s post hoc tests *p < 0.05, **p < 0.01, ***p < 0.001 In Panel **b**, statistical differences between the F1S and F2S formulations are indicated on data collected from day 120 through 180, **p < 0.01. For statistical differences of OF vs F1S and OF vs F2S from day 120 through 180, all *p* values are <0.001 and not shown in Fig. 5b.

Films containing 1 × 10^12^ virus genomes within either the high-viscosity (F1S) or low-viscosity (F2S) film matrix and stored in controlled environmental chambers held at 4 °C/40–50% RH maintained 100% of their original titer for 150 days (Fig. 5a). Preparations containing the same amount of vector stored in the Standard Control Formulation displayed a significant drop to 97.7 ± 0.1% of the original infectious titer after 2 months (*p* = 0.0001 with respect to F1S and *p* = 0.0003 with respect to F2S) and gradually fell to 90.6 ± 0.2% at the end of the study (*p* = 0.0005 with respect to F1S and F2S). Analysis of data collected from vectors stored in this formulation during the 30–150 day time interval revealed that infectious titer dropped at a rate of 0.086% of the original dose per day. When stored at 25 °C, the infectious titer of the same preparation fell to 93 ± 0.1% of the original titer by day 7, to 80.9 ± 0.3% by day 21, 71.7 ± 1.8% by day 120, and was undetectable after 5 months (Fig. 5b). There was no significant difference between the high (F1S) and low (F2S) viscosity formulations for the first 30 days at 25 °C with approximately 96% of the original dose found in films prepared from each formulation (*p* = 0.074–0.9989, day 0–30). During this time, AAV in the Standard Control Formulation lost 0.7562% of the original potency per day while the rate of degradation of vector in the F1S formulation was sevenfold less (0.1431% per day). That of the F2S formulation followed a similar trend (0.1453%). After 150 days, the amount of vector remaining in films prepared with the high-viscosity formulation (F1S) was significantly higher than that in those prepared with the low-viscosity matrix (F2S, 90.6 ± 0.2% vs. 87.2 ± 0.2%, *p* = 0.001). By the end of the 6 month time period, the amount of vector present in the F2S formulation dropped to 84.7 ± 0.4% while that of the F1S material remained at 90% of the original dose (*p* = 0.002). Films prepared in the F1S formulation also fully preserved vector potency during 16 freeze-thaw cycles that involved 24 h alternating intervals of storage at −80 and 20 °C (Fig. 5c).

### In vivo performance of AAV9 in films: short-term storage at 4 °C

**Fig. 6:**
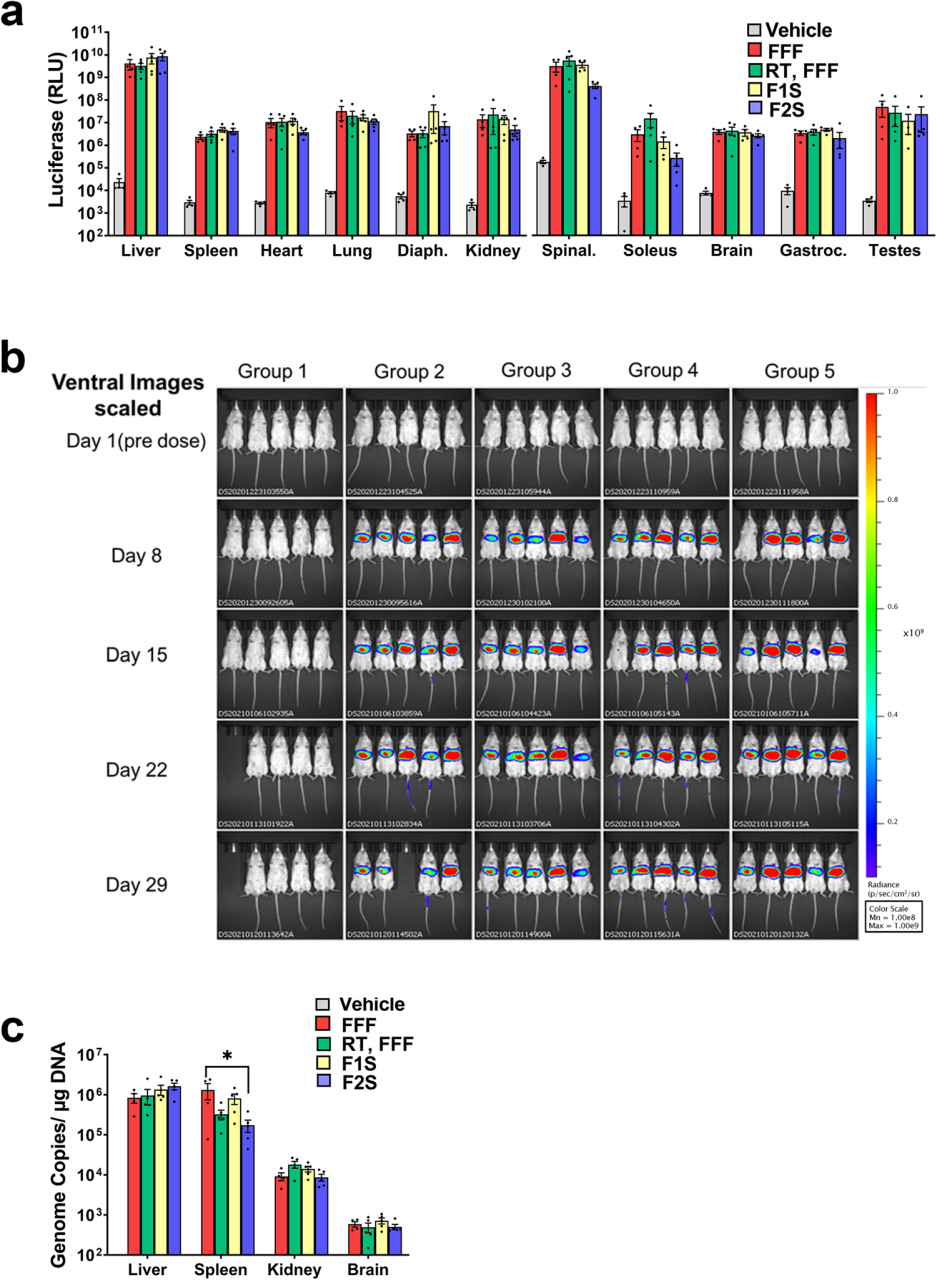
In vivo performance of AAV stabilized in a thin film for 30 days at 4 °C. Mice were given 1.5 × 10^11^ virus genomes of formulated AAV9 CBA-Luc by tail vein injection. **a** Thirty days after administration, animals were sacrificed, organs harvested, and assessed for luciferase transgene expression with bioluminescence imaging. Mean (±standard error of the mean) values for total flux for each organ collected from five mice per treatment group are shown. **b** Bioluminescent images of mice from each treatment group at 1, 8, 15, 22, and 29 days. Relative bioluminescence intensity is shown in pseudo-color, with red and blue representing the strongest and weakest photon fluxes, respectively. NOTE: Two animals, one from the saline control group (Group 1) and that which was given freshly purified vector in the Standard Control Formulation (Group 2), were found deceased in their cages on day 22 and day 29, respectively, with no gross abnormalities observed externally nor internally upon visual inspection of the organs. These deaths were attributed to the blood collection technique that was used at the time. The procedure was immediately changed when these observations were noted so that all mice in the remaining treatment groups were able to complete the full course of the study. Treatment Groups: Group 1: Vehicle (saline control); Group 2: FFF, Group 3: RT, FFF, Group 4: F1S, Group 5: F2S. **c** Organs with high transgene expression were further analyzed for virus genome copies by quantitative real-time PCR. Data reflect the mean (±standard error of the mean) values obtained from tissues collected from five mice per treatment group. Significant differences between animals treated with vectors in the standard control (FFF) and the F1S and F2S formulations were evaluated by one-way ANOVA with Dunnett’s multiple comparison tests. *p < 0.05. Abbreviations (Panels **a** and **c**): Vehicle: saline control, FFF frozen standard control formulation, RT, FFF freshly purified vector in standard control formulation held at room temperature (RT) during the time needed for film formation to be complete then refrozen, F1S high-viscosity film formulation, F2S low-viscosity film formulation, Diaph diaphragm, Spinal Spinal cord, Gastroc gastrocnemius muscle.

When AAV stability within each film matrix at 4 °C seemed promising, an aliquot of films stored at 4 °C for 30 days were rehydrated and administered at a dose of 1.5 × 10^11^ vector genomes by tail vein injection to B6(Cg)-Tyrc-2J/J (B6 Albino) mice. Transgene expression was compared to groups given the same dose of vector stored frozen in Standard Control Formulation (FFF) and an aliquot of the same vector thawed and left at room temperature during the film-forming process (temperature control, RT, FFF, Fig. 6). Quantitative assessment of the luciferase transgene in organs collected 30 days after the administration revealed that vector stabilized in both the F1S and F2S formulations transduced all organs to the same degree as vector stored frozen in the Standard Control Formulation (Fig. 6a, Supplementary Data 1, and Supplementary Data 2). Transgene expression in the liver was 2 log units higher than that in other organs. This was also evident during IVIS imaging during the course of the study, where transgene expression peaked in the liver by day 22 (Fig. 6b). Analysis of key organs (liver, kidney, spleen, and brain) by quantitative real-time PCR revealed that the biodistribution of virus genomes was in line with transgene expression except for the spleen where the number of genome copies present in samples isolated from mice given vector in the F2S formulation was significantly lower than samples from mice given the vector frozen in the Standard Control Formulation (2.8 ± 1.0 × 10^5^ vg/µg DNA vs. 1.3 ± 0.6 × 10^6^ vg/µg DNA, *p* = 0.0287, Fig. 6c).

### In vivo performance of AAV9 in films: long-term storage at 4 °C

**Fig. 7:**
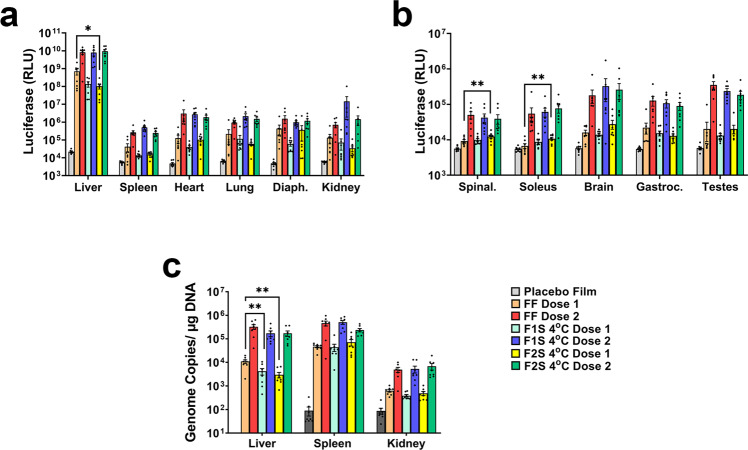
AAV stabilized in a thin film for 150 days at 4 °C performs in vivo in a dose-dependent manner equivalent to that of a frozen vector. Films prepared with high (F1S) and low (F2S) viscosity formulations were rehydrated and diluted with saline to administer 1 × 10^10^ (Dose 1) or 1 × 10^11^ (Dose 2) virus genomes of AAV9 CBA-Luc by tail vein injection. **a**, **b** Luciferase transgene expression in target organs 30 days after administration assessed by bioluminescence imaging. Mean (±standard error of the mean) values for total flux for each organ are shown. Significant differences between animals treated with each dose of the standard control formulation (FF) and the equivalent dose of FS1 and FS2 formulations were evaluated by one-way ANOVA with Dunnett’s multiple comparison tests. **c** Genome copies of AAV9 in organs with high transgene expression as determined by quantitative real-time PCR. Data reflect the mean (±standard error of the mean) values for each treatment group. Significant differences between subgroups with respect to dose were evaluated by one-way ANOVA with Tukey’s multiple comparison tests. **a**, **b** Diaph diaphragm, Spinal spinal cord, Gastroc gastrocnemius muscle *p < 0.05, **p < 0.01.

In a follow-up study, AAV stored in each film formulation at 4 °C for 150 days was shipped without cold packs/dry ice from Austin, Texas to Durham, NC. Temperature monitoring data for this study is depicted can be found in Supplemental Fig. 2. Vector prepared in the Standard Control Formulation was shipped frozen on dry ice at the same time. Films were rehydrated and vector administered by tail vein injection to mice at two different doses (1 × 10^10^ vg, Dose 1 and 1 × 10^11^ vg, Dose 2) to determine a dose effect and minimize potential saturation of the luciferase transgene in target tissues like the liver. As in the previous study, quantitative assessment of the luciferase transgene in organs collected 30 days after administration revealed that there was no significant difference in transgene expression achieved in all tissues of animals given vector in the frozen Standard Control Formulation and that stabilized in either film formulation at the high dose (Dose 2, Fig. 7a, b, FF vs. F1S, *p* = 0.0862–0.9914; FF vs. F2S *p* = 0.5345–0.9981). A significant increase in transgene expression in the spinal cord (*p* = 0.0097) and the soleus (*p* = 0.006) was noted in mice given the low dose of vector stabilized in the F2S formulation with respect to those given the same dose of vector in the standard frozen liquid control formulation (FF, Dose 1, Fig. 7b). Livers from mice given the lowest dose of AAV (Dose 1) in either film formulation contained a significantly lower number of virus genomes than those from mice given the same dose of vector stored in the frozen Standard Control Formulation (F1S *p* = 0.0067, F2S *p* = 0.0016, Fig. 7c). This only resulted in a significant reduction in hepatic transgene expression in mice given the F2S formulation (*p* = 0.0256, F2S, Dose 1, Fig. 7a).

### In vivo performance of AAV9 in films: long-term storage at 25 °C

**Fig. 8:**
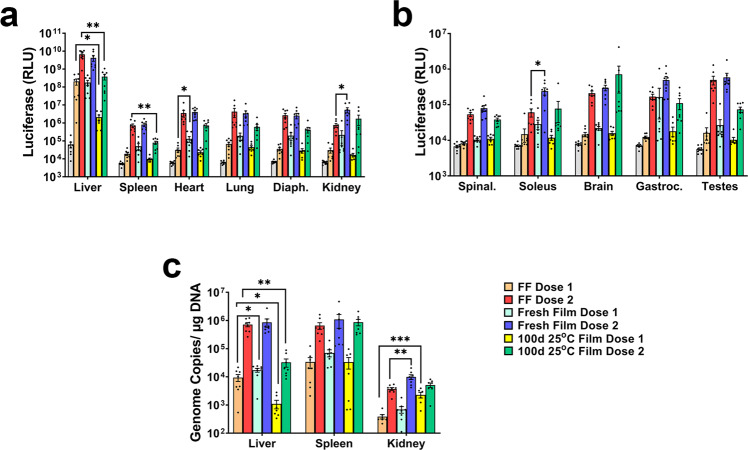
AAV stabilized in a thin film for 100 days at room temperature induces transgene expression in a dose-dependent manner. Freshly prepared and aged films created with the high-viscosity formulation were rehydrated and diluted with saline to administer 1 × 10^10^ (Dose 1) or 1 × 10^11^ (Dose 2) virus genomes of AAV9 CBA-Luc by tail vein injection. **a**, **b** Luciferase transgene expression in target organs 30 days after administration assessed by bioluminescence imaging. Mean (±standard error of the mean) values for total flux for each organ are shown. Significant differences between animals treated with each dose of the Standard Control Formulation (FF) and the equivalent dose of fresh films and 100-day old films made from the F1S  formulation were evaluated by one-way ANOVA with Dunnett’s multiple comparison tests. **c** Organs with high transgene expression were further analyzed for virus genome copies by quantitative real-time PCR. Data reflect the mean (±standard error of the mean) values for each treatment group. Significant differences between subgroups with respect to dose were evaluated by one-way ANOVA with Dunnett’s multiple comparison tests. **a**, **b** Diaph diaphragm, Spinal spinal cord, gastroc gastrocnemius muscle *p < 0.05, **p < 0.01, ***p < 0.001.

In a final proof-of-principle study, the transduction efficiency of AAV stabilized in films prepared with the F1S formulation and stored at 25 °C for 100 days was compared to that of films freshly prepared as well as vector stored frozen in the Standard Control Formulation (Fig. 8). Films were shipped without cold packs/dry ice from Austin, Texas to Durham, NC and temperature within the package monitored throughout transit (Supplemental Fig. 3). Vector prepared in the Standard Control Formulation was shipped frozen on dry ice at the same time. Upon arrival, films were rehydrated and the vector was administered by tail vein injection to mice at two different doses (1 × 10^10^ vg, Dose 1 and 1 × 10^11^ vg, Dose 2). The F1S formulation was selected for this study due to its superior performance with respect to the F2S formulation when stored at 25 °C (Fig. 5b). Quantitative assessment of the luciferase transgene in organs collected 30 days after administration revealed that there was no significant difference in transgene expression in tissues from animals given vector in freshly made films and the frozen Standard Control Formulation at both doses (FF, Fig. 8a, b, FF Dose 1 vs Fresh Film Dose 1, *p* = 0.0747–0.9237; FF Dose 2 vs Fresh Film Dose 2 *p* = 0.0778–0.9921). An exception to this was that there was significantly more luciferase expression in the kidney (5.36 ± 1.96 × 10^6^ RLU, *p* = 0.0328) and soleus (2.41 ± 0.63 × 10^5^ RLU, *p* = 0.0226) after administration of the highest dose (Dose 2) with respect to that observed in kidneys (7.47 ± 2.21 × 10^5^ RLU) and soleus (6.06 ± 1.60 × 10^4^ RLU) from mice given vector in the frozen Standard Control Formulation. The number of virus genomes isolated from the kidneys of each treatment group followed the same trend (9.95 ± 1.9 × 10^3^ vg/µg DNA, fresh film vs. 3.74 ± 0.43 × 10^3^ vg/µg DNA, FF, Fig. 8c). Transgene expression in tissues obtained from animals given vector prepared in films stored at 25 °C for 100 days was slightly reduced or remained unchanged for most tissues with respect to those obtained from mice given, vector stored in the frozen Standard Control Formulation at each dose tested (Fig. 8a, b). However, a drop in RLU of 2 log units (*p* = 0.0314) was noted in the liver of animals given the low dose of the vector from 100-day films, while those given the highest dose demonstrated a drop of 1.2 log units in RLU (*p* = 0.0018) with respect to those given vector stored frozen in Standard Control Formulation (Fig. 8a). The number of virus genomes in livers from these animals followed the same trend (Fig. 8c). While a significant decrease in transgene expression was also found in the spleens of animals given the high dose of a vector from 100-day old films (*p* = 0.0077, Fig. 8a), the number of vector genomes present in these tissues were not statistically different from those given the same dose of vector in the standard control formulation (Fig. 8c).

## Discussion

Robust stability-indicating assays are key to any clinical gene therapy program^37^. For AAV vectors, these have traditionally fallen into two classifications: those that characterize the number of physical particles or genomes present in a preparation and those that assess vector potency^38–41^. To date, PCR-based assays are the most widely used and accepted method for quantification of AAV vectors as they are simple and robust under ideal conditions^42–44^. However, the number of virus genomes present in a given production lot, cell, or tissue sample does not fully demonstrate that the virus is functional. Thus, assays that can demonstrate the ability of the vector to enter target cells and/or tissues (physical transduction) and efficiently express the encoded transgene cassette (functional transduction) are preferred when screening formulations that promote the stability of AAV at elevated temperatures. In the studies summarized here, we first assessed the transduction efficiency of the AAV9 vector through a serial dilution assay to determine where luciferase transgene expression declined linearly and was not saturated nor impacted by components of the film matrix. Parallel studies were performed on cell lysates to also determine the number of viral genomes taken up by cells at the same concentrations. In the short-term assessment of the physical stability of AAV9 in the Standard Control Formulation at 25 °C, transgene expression declined while the number of virus genomes remained constant throughout the 7-day period (Fig. 1). This observation has also been shared by others and can be attributed to the presence of extraneous single-stranded DNA released from capsids that were ruptured during storage but could enter cells through non-specific mechanisms or from unstable capsids that could enter cells but not support the additional steps (e.g. endosomal escape, nuclear entry, etc.) required for successful transgene expression^45–47^. In contrast, the biodistribution of virus genomes in target organs correlated well with transgene expression at each of the doses tested in mice (Figs. 6–8). This illustrates a clear difference between in vitro assays which can support compromised capsids and vector genomes to enter cells due to close proximity in a culture dish where they are most likely cleared from the circulation before reaching a target tissue in vivo. Further refinement of the in vitro assay to minimize the presence of extracapsular genomic DNA and time for compromised viruses to enter cells is warranted for future studies and is currently underway in our laboratories.

The Standard Control Formulation for AAV9 storage utilized in these studies consists of a hyperosmotic solution (1321 mOsm). Preparations to be given by the intravenous route should generally be under 1000 mOsm for small volume (<100 ml) and 500 mOsm for large volume (>100 ml) preparations to minimize irritation, the sensation of heat, and pain upon administration^30^. While hyperosmotic solutions such as the standard control formulation under evaluation here have been documented to minimize aggregation and loss of potency of AAV vectors during freeze-thaw and in the frozen state^12,17^, they stimulate aggregation when stored unfrozen at 25 °C (Supplemental Fig. 4). In this study, polymers were screened to reduce the overall viscosity of the film matrix so that the final product could be rehydrated and administered by intravenous injection (Fig. 2a). While we successfully accomplished this goal, we also found that the F1S formulation offered superior stability to the AAV9 vector at 25 °C (Fig. 5b). This is due to the ability of that polymer to interact with other formulation components to bind to the vector and preserve the three-dimensional shape of capsids at elevated temperatures^35^. The binding capacity of the polymer with the vector was also illustrated by the slower release rate obtained by the F1S formulation with respect to the low-viscosity F2S formulation (Fig. 3) and the additional steps required to release AAV capsid proteins from the film matrix prior to resolution on an SDS-PAGE gel (Supplemental Fig. 5). While somewhat viscous, the F1S preparation was only mildly hyperosmotic (386 ± 30 mOsm), suggesting that viscous formulations may offer an advantage over hyperosmotic formulations in improving AAV stability at temperatures greater than 4 °C as long as they can be administered with minimal resistance from the injection device and are well tolerated by the patient^48,49^. This specific polymer was selected for use in the previous formulation developed for recombinant adenovirus for its superior film-forming and mucoadhesive properties to improve the potency of vaccines given by oral and nasal routes, for which the platform was specifically designed^24–26^. Hydrogels and microneedles prepared from this polymer have been administered by the subcutaneous and intramuscular routes for sustained release of biologic and small molecule drugs over time^50–52^. While the polymer has been safely administered by the intravenous route, administration of repeat doses for some gene therapy applications has motivated a further refinement of the film matrix^53^. Formulation of vector at a concentration of 10 times that needed for the low dose treatment groups used in the in vivo studies summarized in Figs. 7 and 8 provided us with an approach that would support repeat dosing of vector and also reduced osmolality of the final preparation (5 ± 1.2 mOsm). Additional work is underway to determine if this would be feasible for doses required for administration to non-human primates and humans.

We and others have found that the final pH of a given formulation reduces AAV stability and transduction efficiency through disruption of the capsid structure, which is somewhat serotype dependent (Fig. 2b)^11,12,17^. We also found that this sensitivity was more profound as films were stored at room temperature over time (Fig. 2c). It has been shown that changes in formulation pH can occur during various drying processes as buffering components become concentrated^11^ and during storage through environmental factors, as illustrated by diffusion of carbon dioxide gas through the packaging of viral vectors shipped on dry ice^54^. A slight drop in pH from 7.5 to 7 was observed for the F1S and F2S formulations during the film-forming process. After this, the pH remained unchanged during the 16 freeze-thaw cycles summarized in Fig. 5c, while that of the Standard Control Formulation dropped from pH 7.4 to 5.0 during the first freeze-thaw and continued to drop to that value during each cycle. It is also important to note that the pH of the thawed Standard Control Formulation began to drift from 7.4 at the start of the study to 6.0 after the sixteenth freeze-thaw cycle.

Surfactants have been a common excipient utilized in AAV formulations to prevent aggregation and to prevent adsorption of vectors to storage containers and surgical devices (Table 1)^7–11,14,55^. In this report, we found that the presence of a surfactant within the film matrix at a concentration that was not cytotoxic (Supplemental Fig. 6) was key in maintaining AAV stability during the film-forming process (Fig. 2e). Work with adenovirus revealed that this zwitterionic compound at the concentration utilized in our formulations assembles in the form of micelles or premicellar aggregates, near or around the surface of virus capsids and that the sugar and polymer base (also used in these studies) force it to form much smaller micelles that coat the capsid surface during the drying process, preserving the native conformation of the capsid and protecting it from inactivation during exposure to thermal and environmental stressors^35^. It is also interesting to note that a sharper distinction between the surfactants with respect to size was observed with adenovirus, with the smallest C8 compound retaining only 86% of transduction efficiency, C12 92%, and the C16 compound 100% after drying. The fact that AAV potency during the film-forming process was not as impacted as that of adenovirus suggests that since the AAV9 capsid also bears a negative charge, compounds bearing carbon side chains shorter in length also could form protective micelles around the smaller AAV particles in a similar manner. Additional studies are clearly warranted to further understand how these compounds interact with different AAV serotypes and how they differ from adenovirus based on side chain length and stability profiles.

Previous studies involving lyophilization of live viruses have shown that the amount of moisture remaining within a solid dosage form can compromise stability and that there is a very narrow window between excess levels that facilitate proteolysis and other degradation pathways and insufficient levels to interact with excipients to maintain capsid integrity during drying^11,21,56–58^. This may also be the case for AAV9 within the film matrix as the high-viscosity F1S preparation retained more water than the low-viscosity F2S preparation yet offered superior stability when stored at 25 °C/60% RH (Figs. 4a, 5b). We believe that the polymer worked in concert with other excipients to protect the vector from direct interaction with water trapped within the film matrix. The environment in which the films were stored also contributed to the improved stability profile, as it has been shown that environmental humidity plays a role in the viability of viruses in nature and those formulated in non-traditional delivery platforms (Fig. 4b)^24,59,60^.

The gold standard for determining vector biodistribution in experimental animals has been the assessment of vector DNA, mRNA, and/or protein in target tissues^61^. Using this approach in the studies summarized here, we found that following intravenous administration, biodistribution patterns of the AAV9 CBA-Luc vector stabilized within the film matrix were similar to those reported in the literature (Fig. 6)^62–66^. Expansion of these studies to include administration of vector at two doses (1 × 10^10^ and 1 × 10^11^ vg/mouse) revealed a notable drop in transgene expression and genome copies present in the livers of animals given the lowest dose of AAV stabilized within the film matrix for 150 days (Fig. 7), lower than that previously reported in a mouse model^63,66^. This suggests that there may not be a linear dose-response relationship with vector stabilized in a film, which may be due to difficulties of releasing smaller amounts of virus from the film matrix (Supplemental Fig. 5). This effect may also be the reason for the observed increase in AAV genomes in the kidneys of mice given film-based preparations as polymer-vector complexes may be trapped in this organ^67^. It is also important to realize that doses from 0.5 × 10^13^ to 1.5 × 10^13^ vg/kg of AAV9 have been utilized in clinical studies and that the FDA-approved product Zolgesma is given at doses of 6.7 × 10^13^ and 1 × 10^14^ vg/kg^9,68^. Thus, a dose of 1 ×10^11^ vg/mouse can be scaled to represent 0.5 × 10^13^ vg/kg, the lowest dose of AAV9 currently used in humans, making our results with the highest of the tested doses more clinically relevant. Biodistribution profiles of vector stored in the F1S film matrix at 25 °C for 100 days were similar to those seen with freshly prepared vector except in the liver and spleen (Fig. 8). While AAV potency in this formulation at this timepoint did decline according to the in vitro assay utilized in these studies, it was not at the same magnitude as that seen in these highly perfused organs which may be more sensitive to changes in potency of preparations given by systemic injection as transduction efficiency in other tissues (heart, lung, brain, kidney, muscle) was not statistically different from that produced by vector stabilized in freshly prepared films or the standard frozen standard control formulation. These observations make it clear that our current in vitro assay is susceptible to overestimation of the potency of preparation as partially disrupted capsids could successfully transduce cells in culture in the presence of a helper virus while they may be more readily cleared in vivo prior to transduction. This motivates us to develop additional stability-indicating assays that assess the physical properties of virus particles during storage that can be used with a modified in vitro potency assay to better predict in vivo transduction patterns in highly perfused organs. We also realize that assessment of in vivo potency of films stored at 25 °C at earlier timepoints would have allowed us to further define the time at which AAV9 could be stored at room temperature without a drop in potency. This is a limitation of this study.

To date, film-based dosage forms have been largely utilized to rapidly administer small molecules into systemic circulation by bypassing the gastrointestinal tract^69^. Only recently has film technology been employed to stabilize small molecules with complex solubility and stability requirements for administration by injection^70,71^. While room temperature formulations for AAV have been described recently^72^, we have further extended those reported shelf lives as well as that of the Standard Control Formulation described in this study with film technology. We have also been the first, to the best of our knowledge, to collect in vivo data with an AAV vector with documented evidence of shipment in the absence of dry ice or cold packs that was successfully administered by injection. This improvement expands the potential for allowing single production lots to be stored in an economical manner until they are needed for those impacted by rare genetic diseases. Although not addressed in this body of work, studies to identify the mechanism by which potency was lost with respect to temperature and relative humidity conditions and to refine the film matrix to further extend the stability profiles beyond what was described here are in progress. A hallmark of AAV vectors is the readily interchangeable profiles seen with and between the various serotypes with respect to vector production, transgene expression, and in vivo transduction. Besides, clinically validated AAV9 used in these experiments, ongoing experiments are pending with respect to the stability of additional AAV serotypes. The outcome of these efforts should facilitate a better understanding of mechanisms of AAV degradation on a molecular level^73,74^ and help identify additional requirements for the successful stabilization of these and other gene therapy vectors in the absence of cold chain requirements.

## Supplementary information

Supplementary Data 1

Supplementary Data 2

Description of Additional Supplementary Files

Supplementary Information

Reporting Summary

## Data Availability

Source data for all figures are available as a single Excel file, Supplementary Data 1. Imaging data can be found in Supplementary Data 2.
