# Supplementary figures and images for: Thermostability and in vivo performance of AAV9 in a film matrix
Source: Commun Med (Lond). 2022 Nov 21;2:148. doi: 10.1038/s43856-022-00212-6 (PMC9681776; doi:10.1038/s43856-022-00212-6)

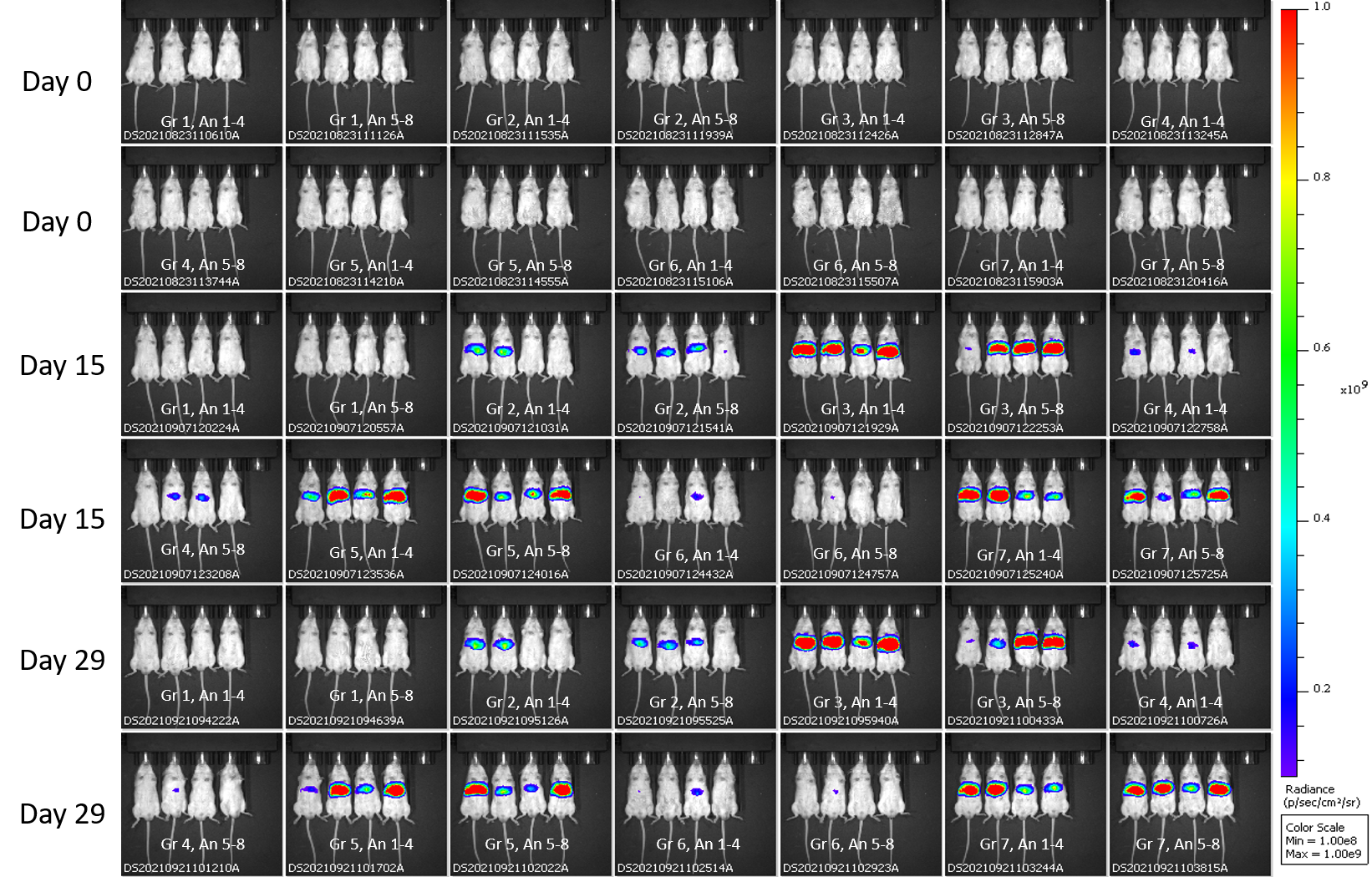

Supplement: Supplementary file 2 — Supplementary Data 2 [file 43856_2022_212_MOESM2_ESM.zip › Fig 7. IVIS images for In vivo study on AAV Stabilized in thin film for 150 days at 4C.png]

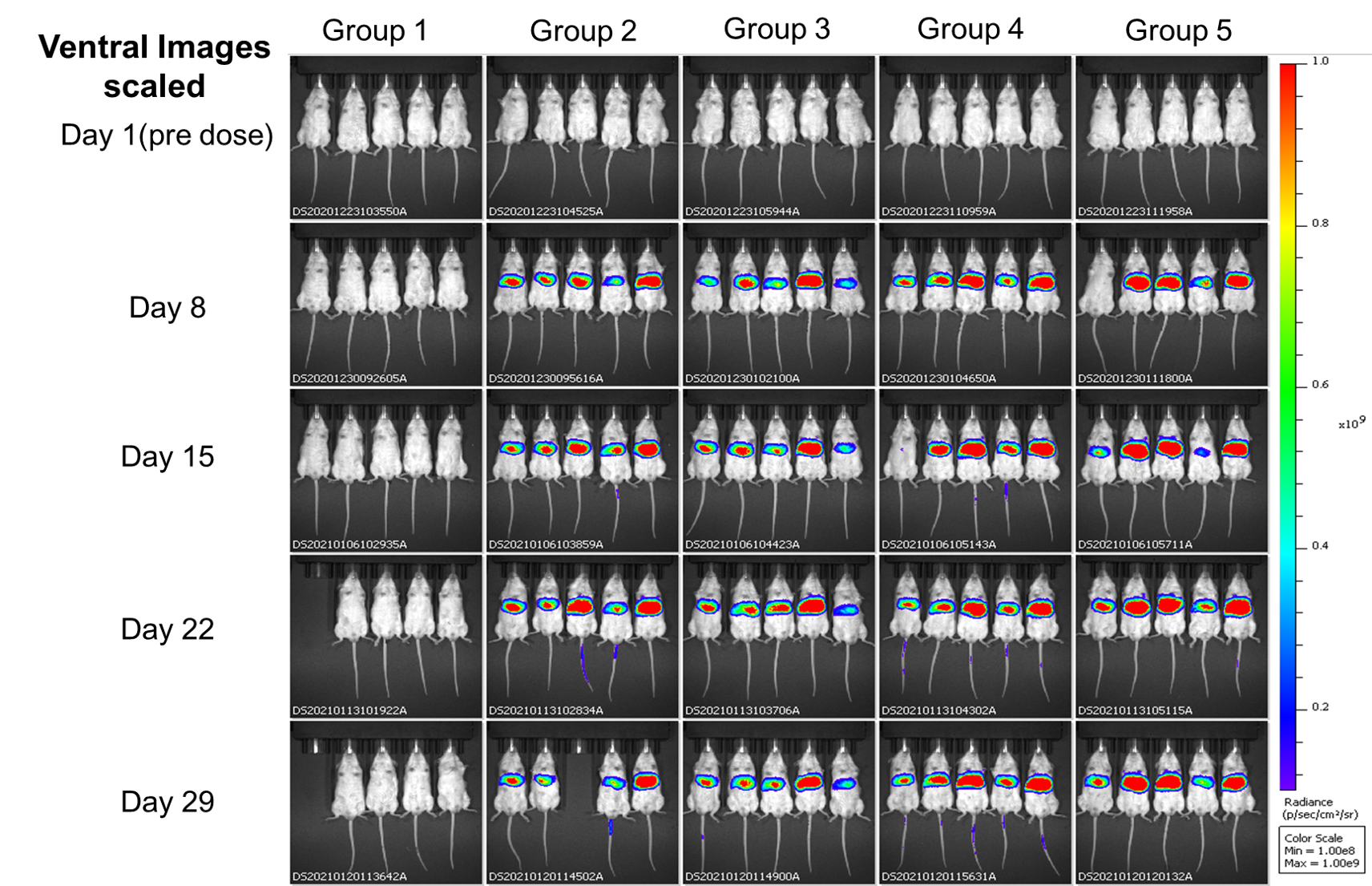

Supplement: Supplementary file 2 — Supplementary Data 2 [file 43856_2022_212_MOESM2_ESM.zip › Fig6. IVIS images for In Vivo Performance of AAV Stabilized in Thin Film for 30 Days at 4°C.png]

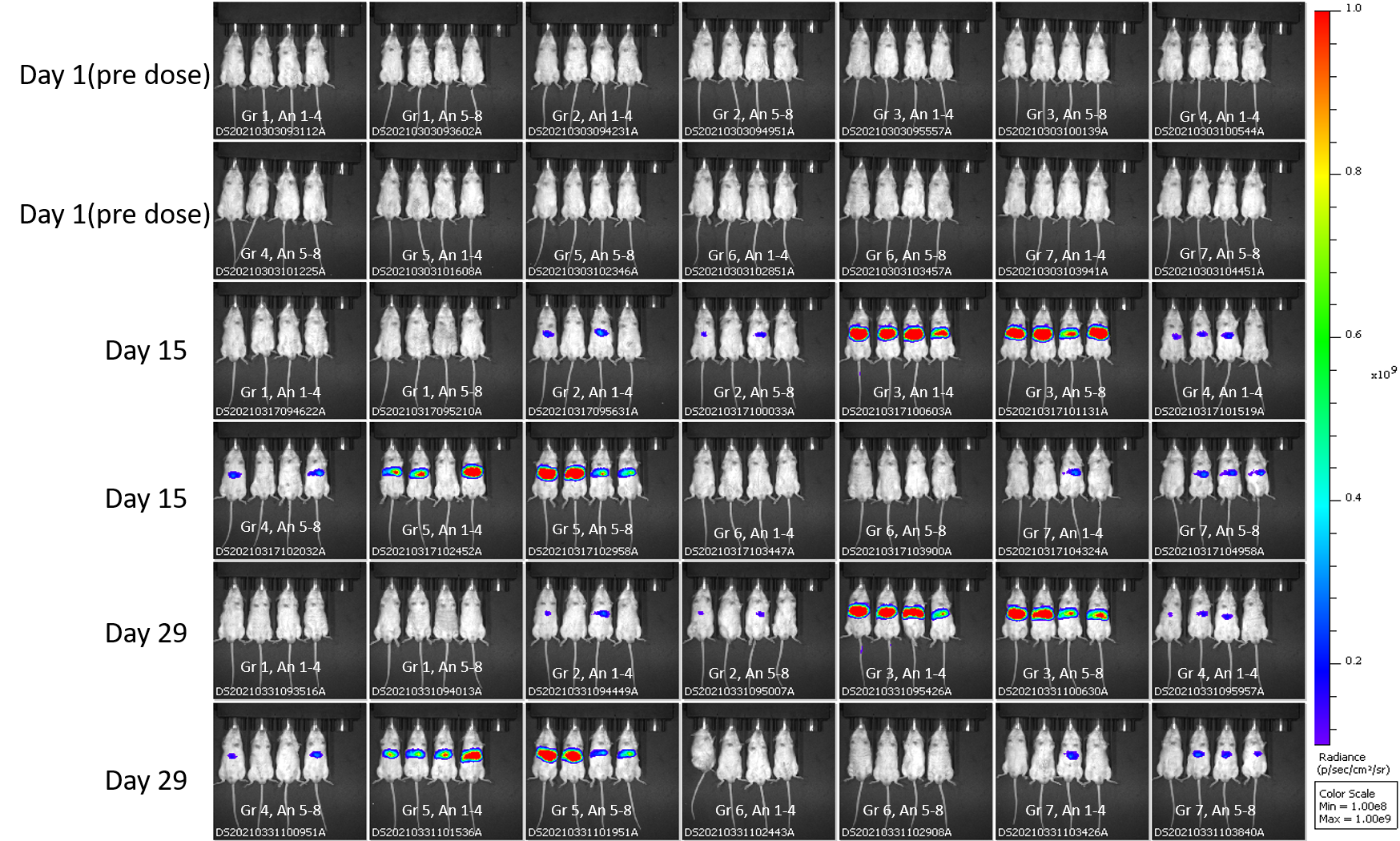

Supplement: Supplementary file 2 — Supplementary Data 2 [file 43856_2022_212_MOESM2_ESM.zip › Fig8. IVIS images for In vivo study on AAV Stabilized in Thin Film for 100 Days at Room Temperature.png]

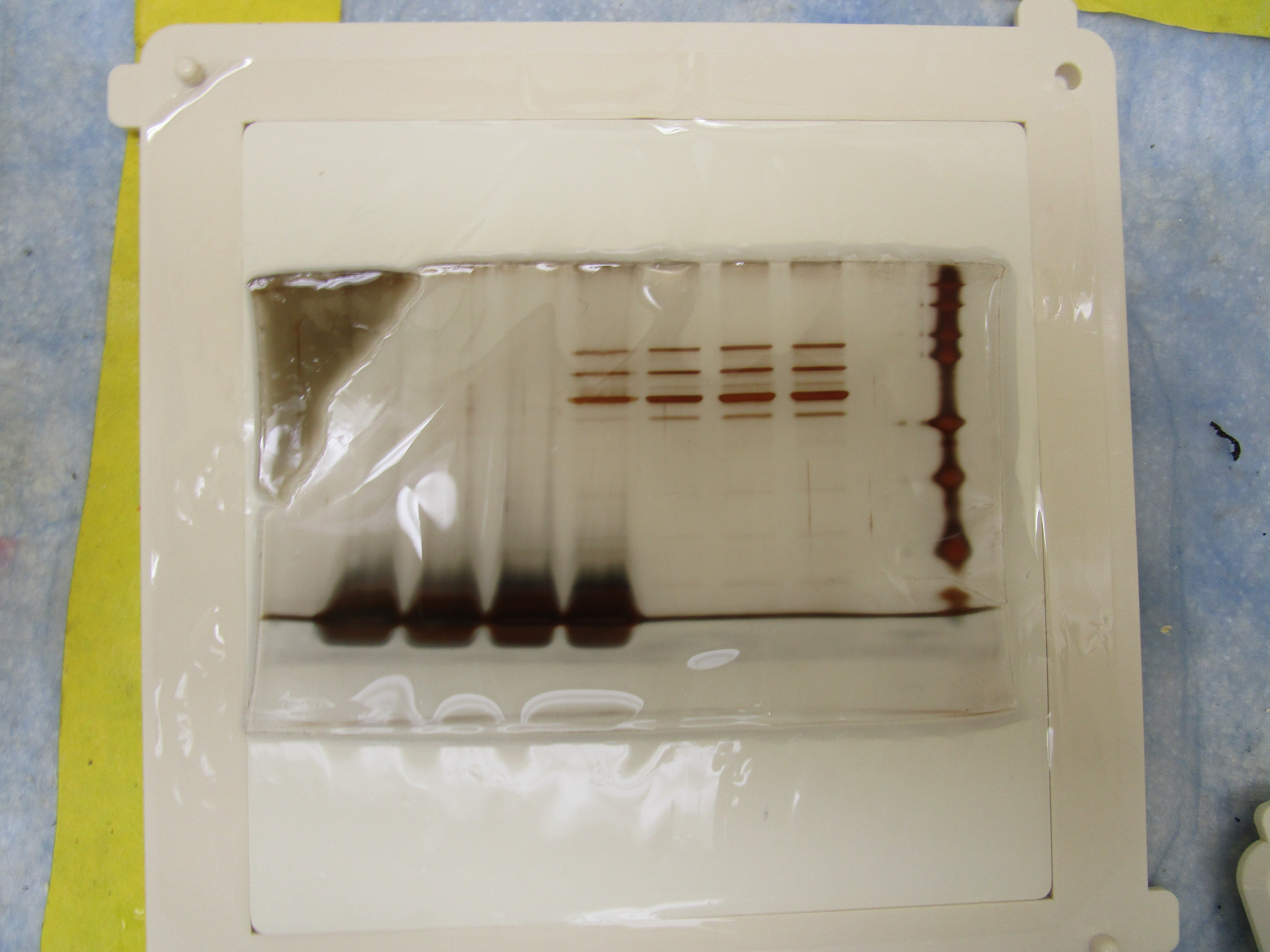

Supplement: Supplementary file 2 — Supplementary Data 2 [file 43856_2022_212_MOESM2_ESM.zip › Original picture for Sup Fig 5.JPG]
