# Supplementary material for: Thermostability and in vivo performance of AAV9 in a film matrix
Source: Commun Med (Lond). 2022 Nov 21;2:148. doi: 10.1038/s43856-022-00212-6 (PMC9681776; doi:10.1038/s43856-022-00212-6)
Supplement: Supplementary file 3 — Description of Additional Supplementary Files [file 43856_2022_212_MOESM3_ESM.pdf]

Description of Additional Supplementary Files

File Name: Supplementary Data 1

Description: Compiled Excel File with Source data for all figures and Supplemental Figures

File Name: Supplementary Data 2

Description: Supplementary Image Files
