# Supplementary material for: Thermostability and in vivo performance of AAV9 in a film matrix
Source: Commun Med (Lond). 2022 Nov 21;2:148. doi: 10.1038/s43856-022-00212-6 (PMC9681776; doi:10.1038/s43856-022-00212-6)
Supplement: Supplementary file 4 — Supplementary Information [file 43856_2022_212_MOESM4_ESM.pdf]

Supplemental Figures

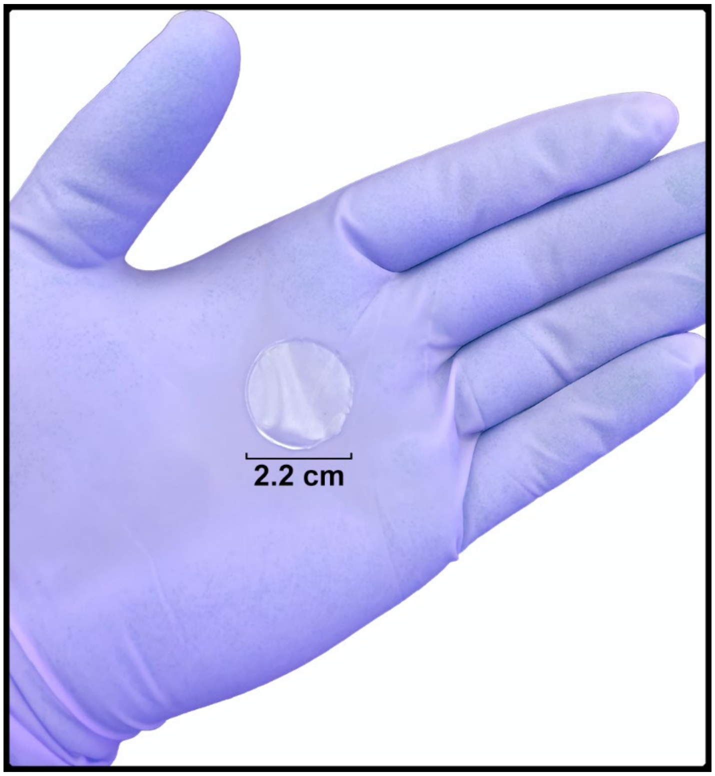

Supplemental Figure 1. Representative Film For *In Vitro* and *In Vivo* Assessment of AAV Thermostability. Clear films have a diameter of 22 mm and an average thickness of 100 ± 9 μm and were prepared under aseptic conditions. They were removed from sterile packaging and rehydrated in a volume of 1 milliliter of culture media for use in infectious titer assays or sterile saline for administration to mice by tail vein injection. **Photo Credit:** Chris Pavlos, The University of Texas at Austin, Jurata Thin Film.

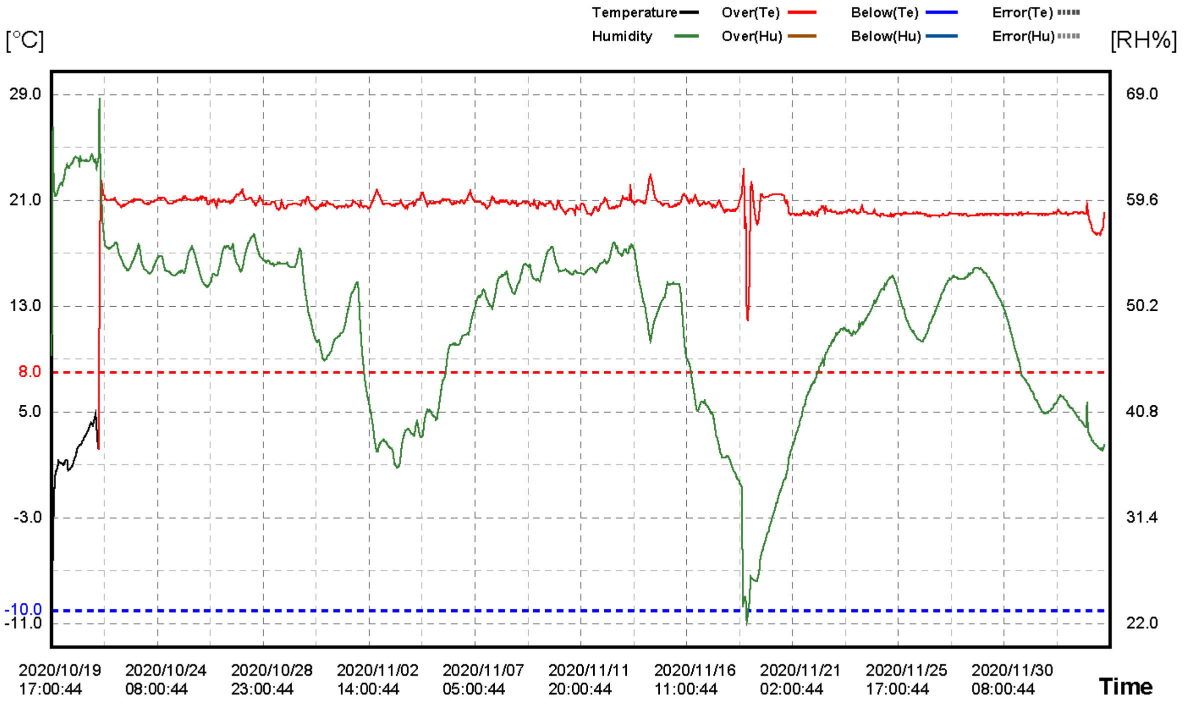

Supplemental Figure 2. Temperature and Humidity Monitoring Data for Films Shipped without Dry Ice/Cold Packs from Austin, Texas to Research Triangle Park, North Carolina after storage for 150 days at 4°C. The red line represents temperature while the green line represents relative humidity measurements. Data was collected with an Ellitech digital datalogging device.

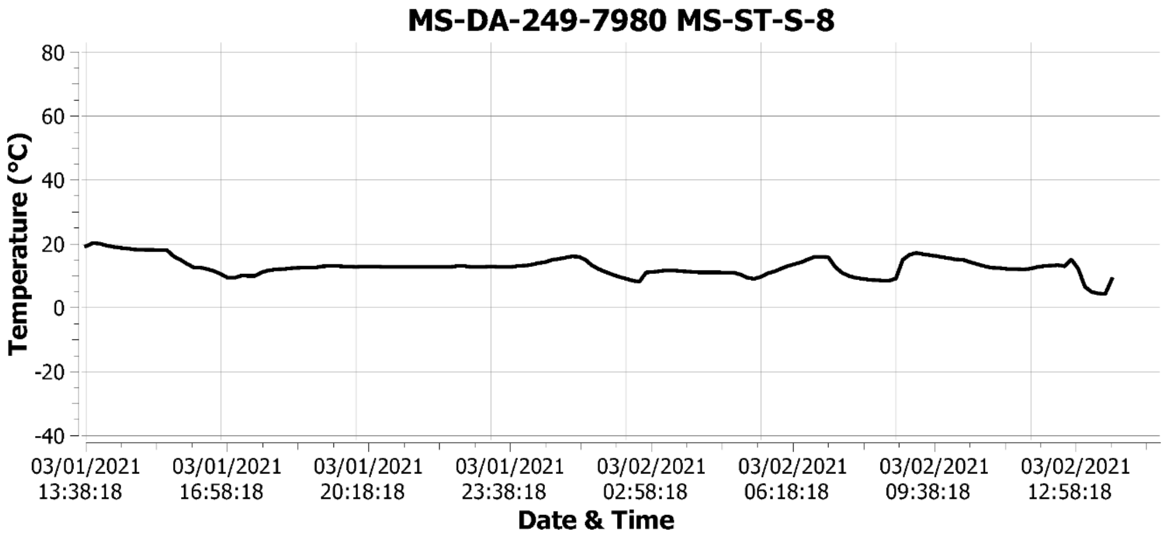

Supplemental Figure 3. Temperature Monitoring Data for Films Shipped without Dry Ice/Cold Packs from Austin, Texas to Research Triangle Park, North Carolina after storage for 100 days at 25°C. Data was collected with an Cryopak iMini USB single use datalogging device.

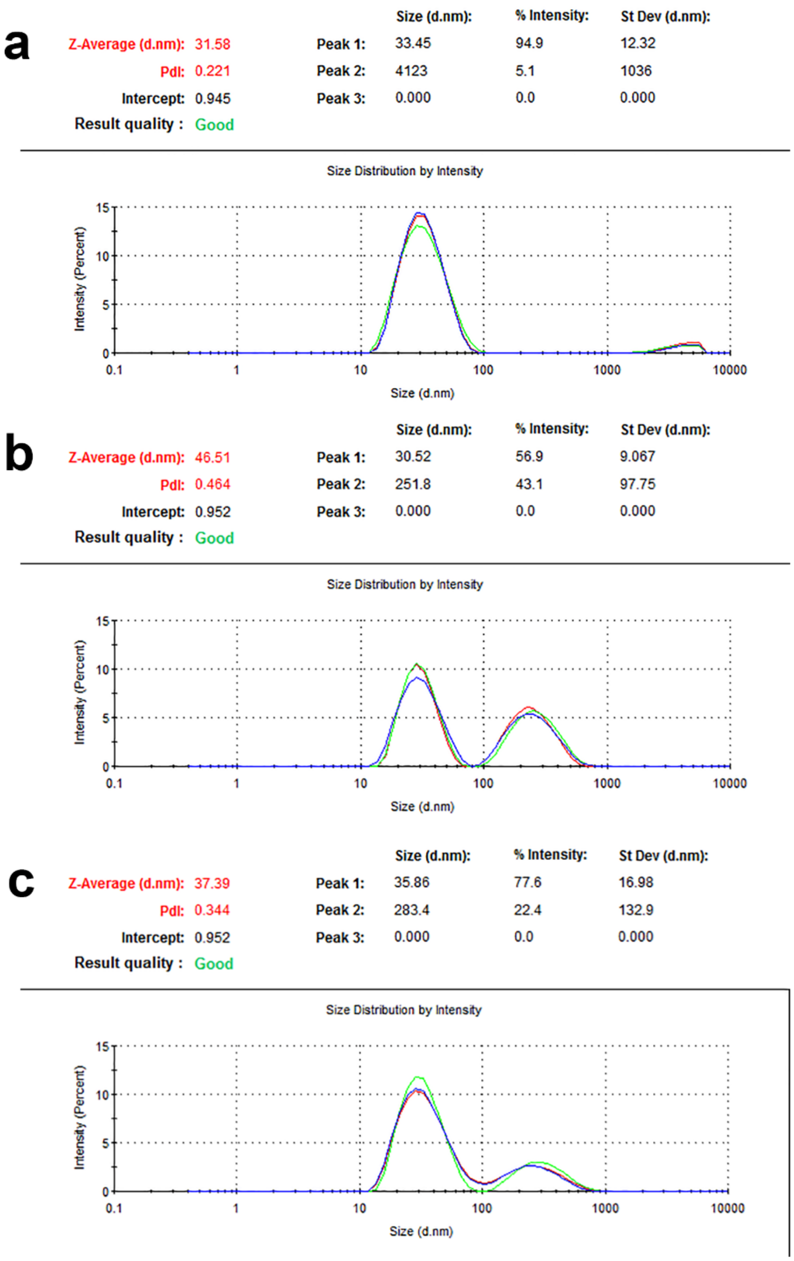

Supplemental Figure 4. Aggregation Phenomena of AAV9 During Storage in a Hyperosmotic Formulation at 25 °C for 7 Days. a. Samples containing 1.0×10^{14} vg of AAV9 in Standard Control Formulation (1,321 mOsm) were removed from -80 °C storage and average particle size assessed upon thaw with a Zetasizer Nano ZS instrument (Malvern Instruments Ltd., Worcestershire, UK). At this time, single AAV particles were uniformly present. b. Particle Size

Distribution after 3 Days at 25 °C. Aggregates of ~283 nm were detected as well as single unaggregated particles. This phenomena continued through the 7 day timepoint (Panel c).

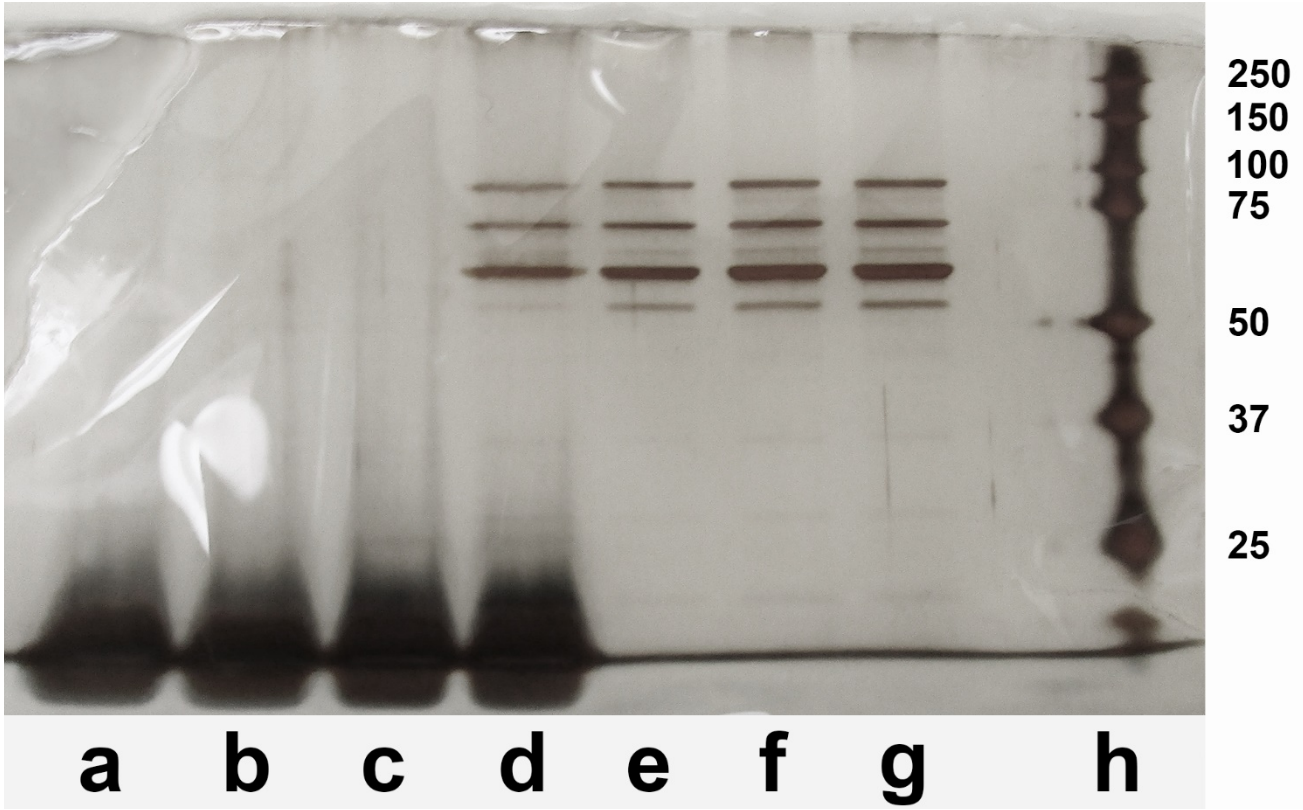

Supplemental Figure 5. AAV Particles are Tightly Bound to Components of the Film Matrix. Samples containing 1.67 x 10^{11} vg of AAV9 were loaded into individual wells of a 10% polyacrylamide gel. Proteins were resolved by electrophoresis at 80V for 30 minutes followed by 120V for an additional 90 minutes. Protein bands were developed by silver staining using the PlusOne Silver Staining Kit (GE Health Science, Uppsala, Sweden). Samples in lanes from left to right were obtained from aliquots of: a. Rehydrated Placebo Film (containing no virus), b. Rehydrated Film Containing AAV, c. Rehydrated Placebo Film after extensive boiling, d. Rehydrated Film containing AAV after extensive boiling. e. Freshly Purified AAV, f. AAV after two freeze-thaw cycles, g. AAV after one freeze-thaw cycle, h. Precision Plus Protein™ Dual Color molecular weight standards (10–250 kDa, Bio-Rad, Hercules, CA). Large bands at the end of Lanes a, c and d consist of the film matrix. The large band and the end of Lane b is comprised of the AAV vector bound within the film matrix.

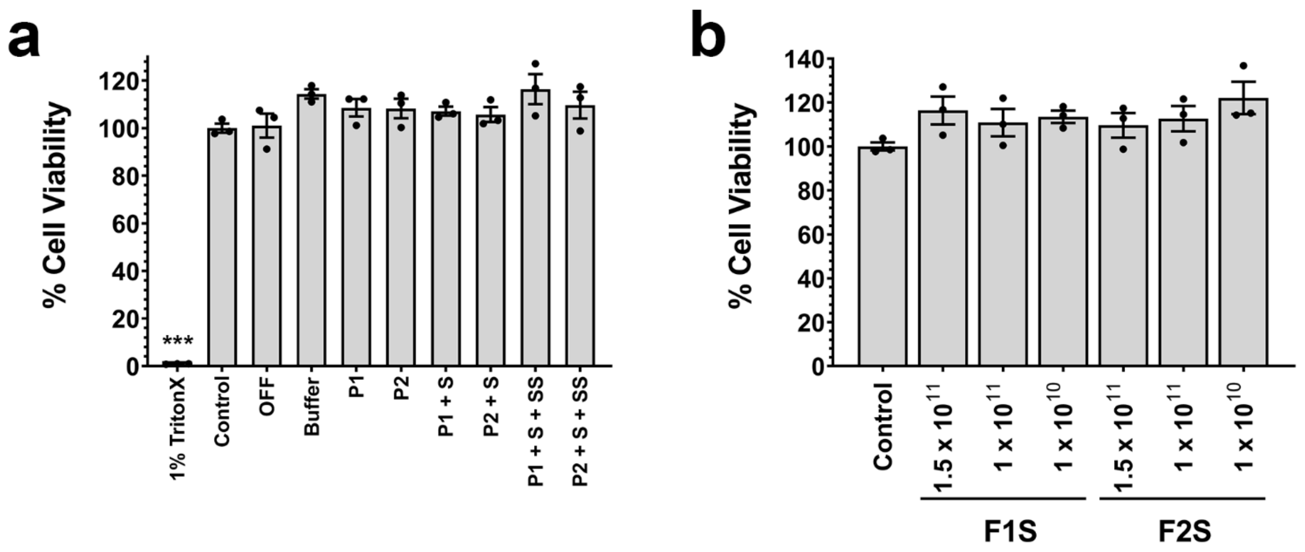

Supplemental Figure 6. Cytotoxicity Profile of AAV in the Presence of Individual Components and Composite Film Formulations (a) and with Different Doses of AAV9 Tested *In Vivo* in the Full Formulation (b). HelaRC32 cells were treated for a period of 2 hours with each preparation containing AAV (1×10^{11} vg Panel A, specified amounts Panel B) and cell viability assessed by measuring the amount of active adenosine triphosphate (ATP) present using a Cell Titer-Glo Luminescent Cell Viability assay (Promega). In each panel, data represent the average ± the standard error of the mean for 3 replicates of each formulation.

Abbreviations: Panel a. OFF: Original Standard Control Formulation (phosphate buffered saline, 350mM NaCl, 5% Sorbitol, 0.001% Pluronic F68, pH 7.4), Buffer: 10 mM Tris buffer pH 8.1, P1: high viscosity polymer base included in the F1S formulation, P2: low viscosity polymer base included in the F2S formulation, S: sugar, SF: surfactant. **Panel b**. F1S: high viscosity complete formulation, F2S: low viscosity complete formulation. **Both Panels.** Control.: Media Control. Results obtained from each treated group were normalized to results obtained from this group representing a fully viable cell population. ***$p < 0.001$

Supplementary Table 1: Summary of Formulations.

	Polymer*							Sugar	Surfactant
	K4M 4,000 cps	A4M 4,000 cps	F4M 4,000 cps	A15C 1,800 cps	A4C 400 cps	K100LV 100 cps	A15LV 18 cps	Sorbitol	PMAL
**Formulation Descriptor**									
**1**	✓							✓	✓
**2**		✓						✓	✓
**3**			✓					✓	✓
**4**				✓				✓	✓
**5**					✓			✓	✓
**6**						✓		✓	✓
**7**							✓	✓	✓
**P**	✓								
**P + S**	✓							✓	
**P + S + SS**	✓							✓	✓
**F1S**	✓							✓	✓
**F2S**						✓		✓	✓

*Values listed under each polymer type represent the viscosity of a 2% w/v solution of each polymer prepared in water as supplied by the manufacturer.
