# Supplementary material for: Thermostability and in vivo performance of AAV9 in a film matrix
Source: Commun Med (Lond). 2022 Nov 21;2:148. doi: 10.1038/s43856-022-00212-6 (PMC9681776; doi:10.1038/s43856-022-00212-6)
Supplement: Supplementary file 5 — Reporting Summary [file 43856_2022_212_MOESM5_ESM.pdf]

nature research

Corresponding author(s): COMMSMED-22-0074A

Last updated by author(s): Oct 7, 2022

Reporting Summary

Nature Research wishes to improve the reproducibility of the work that we publish. This form provides structure for consistency and transparency in reporting. For further information on Nature Research policies, see our Editorial Policies and the Editorial Policy Checklist.

Statistics

For all statistical analyses, confirm that the following items are present in the figure legend, table legend, main text, or Methods section.

n/a	Confirmed	
☐	☒	The exact sample size (*n*) for each experimental group/condition, given as a discrete number and unit of measurement
☐	☒	A statement on whether measurements were taken from distinct samples or whether the same sample was measured repeatedly
☐	☒	The statistical test(s) used AND whether they are one- or two-sided<br>*Only common tests should be described solely by name; describe more complex techniques in the Methods section.*
☐	☒	A description of all covariates tested
☐	☒	A description of any assumptions or corrections, such as tests of normality and adjustment for multiple comparisons
☐	☒	A full description of the statistical parameters including central tendency (e.g. means) or other basic estimates (e.g. regression coefficient) AND variation (e.g. standard deviation) or associated estimates of uncertainty (e.g. confidence intervals)
☐	☒	For null hypothesis testing, the test statistic (e.g. *F*, *t*, *r*) with confidence intervals, effect sizes, degrees of freedom and *P* value noted<br>*Give P values as exact values whenever suitable.*
☒	☐	For Bayesian analysis, information on the choice of priors and Markov chain Monte Carlo settings
☒	☐	For hierarchical and complex designs, identification of the appropriate level for tests and full reporting of outcomes
☒	☐	Estimates of effect sizes (e.g. Cohen's *d*, Pearson's *r*), indicating how they were calculated

Our web collection on statistics for biologists contains articles on many of the points above.

Software and code

Policy information about availability of computer code

Data collection	*Provide a description of all commercial, open source and custom code used to collect the data in this study, specifying the version used OR state that no software was used.*
Data analysis	*Provide a description of all commercial, open source and custom code used to analyse the data in this study, specifying the version used OR state that no software was used.*

For manuscripts utilizing custom algorithms or software that are central to the research but not yet described in published literature, software must be made available to editors and reviewers. We strongly encourage code deposition in a community repository (e.g. GitHub). See the Nature Research guidelines for submitting code & software for further information.

Data

Policy information about availability of data

All manuscripts must include a data availability statement. This statement should provide the following information, where applicable:

- Accession codes, unique identifiers, or web links for publicly available datasets
- A list of figures that have associated raw data
- A description of any restrictions on data availability

The authors confirm that the data supporting the findings of this study are available within the article and its supplementary materials.

Field-specific reporting

Please select the one below that is the best fit for your research. If you are not sure, read the appropriate sections before making your selection.

☒ Life sciences ☐ Behavioural & social sciences ☐ Ecological, evolutionary & environmental sciences

For a reference copy of the document with all sections, see nature.com/documents/nr-reporting-summary-flat.pdf

Life sciences study design

All studies must disclose on these points even when the disclosure is negative.

Sample size	For studies involving mice, we have found (both in the literature and from personal experience) that a 20% change in gene expression would be a statistically significant event. Thus, the minimum number of animals needed per treatment group for these studies was determined by the following formula for a simple analysis of variance: n = 2(Za + Zß)2σ^2/Δ^2 Where n is the sample size, Za is the value for the is the value for the two-tailed a, Zß is the value for the one-tailed ß, σ is the sample standard deviation, and Δ is the smallest difference between the two study groups thought to be important. Values used in the equation were: Za=1.96, Zß = 1.282, σ = 0.1, Δ = 0.25 to yield an n of 4.3, so an n of 5 animals per group was chosen. The same process was followed for all other studies summarized in the manuscript.
Data exclusions	No data was excluded from any of the analyses of the datasets included in the manuscript.
Replication	In vitro studies (aside from the long term stability studies) were performed in triplicate. In vivo studies were performed once.
Randomization	Films and formulations were prepared in batch for each studies and replicates at each timepoint were randomly selected.
Blinding	Blinding of specific formulations was performed using the nomenclature in the figures and figure legends of the manuscript so that technicians and data analysts were blinded.

Reporting for specific materials, systems and methods

We require information from authors about some types of materials, experimental systems and methods used in many studies. Here, indicate whether each material, system or method listed is relevant to your study. If you are not sure if a list item applies to your research, read the appropriate section before selecting a response.

Materials & experimental systems

n/a	Involved in the study	
☒	☐	Antibodies
☐	☒	Eukaryotic cell lines
☒	☐	Palaeontology and archaeology
☐	☒	Animals and other organisms
☒	☐	Human research participants
☒	☐	Clinical data
☒	☐	Dual use research of concern

Methods

n/a	Involved in the study	
☒	☐	ChIP-seq
☒	☐	Flow cytometry
☒	☐	MRI-based neuroimaging

Eukaryotic cell lines

Policy information about cell lines

Cell line source(s)	All cell lines were either proprietary (to make AAV) or purchased from ATCC
Authentication	Not performed.
Mycoplasma contamination	All cell lines were mycoplasma free (as determined by PCR and clinical testing)
Commonly misidentified lines (See ICLAC register)	None.

Animals and other organisms

Policy information about studies involving animals; ARRIVE guidelines recommended for reporting animal research

Laboratory animals	Male B6(Cg)-Tyrc-2J/J (B6 Albino) mice (8-10 weeks) were obtained from the Jackson Laboratory (Bar Harbor, ME)
Wild animals	None

Field-collected samples	None
Ethics oversight	All procedures were approved by the Institutional Animal Care and Use Committee of Mispro Biotech Services (Research Triangle Park) and are in accordance with the guidelines established by the National Institutes of Health for the humane treatment of animals.

Note that full information on the approval of the study protocol must also be provided in the manuscript.
